# Computational Study of the Ion and Water Permeation and Transport Mechanisms of the SARS-CoV-2 Pentameric E Protein Channel

**DOI:** 10.3389/fmolb.2020.565797

**Published:** 2020-09-23

**Authors:** Yipeng Cao, Rui Yang, Wei Wang, Imshik Lee, Ruiping Zhang, Wenwen Zhang, Jiana Sun, Bo Xu, Xiangfei Meng

**Affiliations:** ^1^Tianjin Medical University Cancer Institute and Hospital, National Clinical Research Center for Cancer, Key Laboratory of Cancer Prevention and Therapy, Tianjin, China; ^2^Tianjin’s Clinical Research Center for Cancer, Tianjin, China; ^3^National Supercomputer Center in Tianjin, TEDA – Tianjin Economic-Technological Development Area, Tianjin, China; ^4^Department of Infection and Immunity, Tianjin Union Medical Center, Nankai University Affiliated Hospital, Tianjin, China; ^5^College of Physics, Nankai University, Tianjin, China; ^6^Center for Intelligent Oncology, Chongqing University School of Medicine and Chongqing University Cancer Hospital, Chongqing, China

**Keywords:** COVID-19, SARS-CoV-2, envelope (E) protein, ion channel, permeation mechanisms, MD simulations

## Abstract

Coronavirus disease 2019 (COVID-19) is caused by a novel coronavirus (SARS-CoV-2) and represents the causative agent of a potentially fatal disease that is a public health emergency of international concern. Coronaviruses, including SARS-CoV-2, encode an envelope (E) protein, which is a small, hydrophobic membrane protein; the E protein of SARS-CoV-2 shares a high level of homology with severe acute respiratory syndrome coronavirus (SARS-CoV). In this study, we provide insights into the function of the SARS-CoV-2 E protein channel and the ion and water permeation mechanisms using a combination of *in silico* methods. Based on our results, the pentameric E protein promotes the penetration of cation ions through the channel. An analysis of the potential mean force (PMF), pore radius and diffusion coefficient reveals that Leu10 and Phe19 are the hydrophobic gates of the channel. In addition, the pore exhibits a clear wetting/dewetting transition with cation selectivity under transmembrane voltage, indicating that it is a hydrophobic voltage-dependent channel. Overall, these results provide structure-based insights and molecular dynamic information that are needed to understand the regulatory mechanisms of ion permeability in the pentameric SARS-CoV-2 E protein channel.

## Introduction

COVID-19 is a severe and highly contagious respiratory illness that was first reported in early December 2019. As of August, 2020, 10s of millions of cases have been confirmed, and 100s of 1000s have died. The World Health Organization (WHO) announced a global pandemic of COVID-19 in March 2020. In addition to the hazards of the disease itself, it has also led to substantial turbulence in international financial markets and may cause serious consequences. COVID-19 is a disease caused by a new coronavirus named SARS-CoV-2. Researchers have speculated that the virus originated from bats and was transmitted to humans through an intermediate host (some type of wildlife). Its symptoms include fever, general malaise, dry cough, shortness of breath, and respiratory distress. Similar diseases, including severe acute respiratory syndrome (SARS) and middle east respiratory syndrome (MERS), which are also caused by coronaviruses, have mortality rates of 10 and 36%, respectively (de Wit et al., 2016). Comparatively, COVID-19 has a mortality rate of 1 to 10% in different countries, but appears to be more contagious than SARS and MERS (Baud et al., 2020; Mahase, 2020; Onder et al., 2020; Zhou et al., 2020).

SARS-CoV-2 is a long positive-sense, long single-stranded (30 kb) RNA virus. The structures of different human coronaviruses (HCoVs) are similar. The viral genome is packed with nucleocapsid (N) proteins, forming a helicoidal nucleocapsid protected by a lipid envelope (Chang et al., 2014). Several viral proteins, including the spike (S), envelope (E), and membrane (M) proteins, are embedded within a lipid envelope (Rota et al., 2003; Zaki et al., 2012). The S, M, and N proteins play important roles in receptor binding and virion budding. For example, the M protein participates in virus germination and interacts with the N and S proteins. The S protein has immune recognition sites and can be used to design vaccines (Ibrahim et al., 2020).

Currently, the importance of the E protein has not been completely elucidated. The E protein maintains its morphology after virus assembly by interacting with the M protein (Vennema et al., 1996; Corse and Machamer, 2000; Mortola and Roy, 2004). When a mutation in the gene encoding the E protein occurs, it promotes apoptosis. According to recent studies, coronaviruses contain a viroporin that self-assembles into a pentameric structure and achieves ion selectivity. When a transmembrane voltage is present, the ion channel characteristics of viroporins are more prominent. In addition, the Asn25Ala and Val18Phe mutations in the SARS-CoV E protein might destroy ion channel activity (Torres et al., 2007). Thus, the E protein may play an important role in regulating the ion equilibrium inside and outside the viral envelope.

The ion channel activity of the E protein is specifically correlated with increased pulmonary damage and edema accumulation. Mice infected with viruses possessing E protein ion channel activity present an extensive disruption of the pulmonary epithelia and edema accumulation (Henderson et al., 2020; Jose and Manuel, 2020). In addition, the overproduction of IL-1β, IL-6, and TNF in the airways of the lungs mediate a proinflammatory response that is significantly increased in the lung parenchyma. Thus, the ion conductivity of the E protein pore may stimulate inflammasome secretion. The overproduction of inflammasomes leads to the occurrence of a “cytokine storm.” The cytokine storm and pulmonary edema caused by SARS-CoV-2 infection are the main pathological factors inducing patient death (Henderson et al., 2020; Jose and Manuel, 2020).

In addition, its evolutionary conservation may be an important cause of viral cross-host infection (Graham et al., 2013; Li et al., 2014; Nieto-Torres et al., 2014).

Although previous studies have suggested that the E protein of coronaviruses such as SARS and MERS oligomerizes and is permeable to ions, the specific mechanism of ion permeability and channel properties remain to be explored due to the lack of a crystal structure for the E protein. Li et al. (2014) determined the structure of the SARS-CoV E protein monomer. Surya et al. (2018) determined the pentameric structure of the SARS E protein using nuclear magnetic resonance (NMR) spectroscopy, which provided strong support for the study of the ion permeability mechanism of the SARS-CoV-2 E protein pentamer.

In the present study, we obtained the amino acid sequence of the SARS-CoV-2 E protein from the National Center for Biotechnology Information (NCBI) database (Wu et al., 2020). A model of the pentameric SARS-CoV-2 E protein was built using the homology modeling method, and the reasonableness of the model was evaluated. Subsequently, μs-level molecular dynamics (MD) simulations were performed to evaluate the stability of the pentamer in the membrane environment. We attempted to use potential mean force (PMF) to determine the permeability of different physiological ions and water molecules in the pores of the E protein pentamer. The characteristics of the pentameric channel were analyzed by combining the channel diffusion coefficient and geometric properties. In addition, computational electrophysiology was applied to produce different transmembrane voltages of the system and reveal the effect of the voltage on the ion permeability of the pentameric E protein.

Many experimental methods used by researchers to characterize the protein dynamics of viruses are available, but the motions of proteins at an atomic scale are usually difficult to delineate (Ode et al., 2012). Overall, studies exploring the mechanisms of the pentameric SARS-CoV-2 E protein not only provide valuable insights into the conduction of the channel but also have important implications for our understanding of the difference between SARS-CoV-2 and other coronaviruses.

## Materials and Methods

### Preparation of the SARS-CoV-2 Pentameric E Protein-Membrane Simulation System

The amino acid sequences of human coronavirus E proteins were downloaded from the National Center for Biotechnology Information (NCBI). Cluster-X software was used to generate the multiple sequence alignment (MSA) of the new human coronavirus and the SARS-CoV E protein. A model of the SARS-CoV-2 E protein was built using the SWISSMODEL web server (Biasini et al., 2014). As the pentameric structure of the SARS E protein [Protein Data Bank (PDB) ID: 5X29] (Surya et al., 2018) is the closest to the SARS-CoV-2 E protein, it was used as a template for building the structure of the SARS-CoV-2 E protein. The Rampage web tool was used to evaluate the rationality of the SARS-CoV-2 E protein model. Then, the model was inserted into a POPE:POPS:POPC = 3:1:1 lipid bilayer which is consistent with experimental method described by Wilson et al. (2004) and Surya et al. (2015) via pre-equilibration. The simulation system was set as a 11 nm × 11 nm × 12 nm cubic box in CHARMM-GUI (Jo et al., 2008), comprising the whole pentameric E protein, 330 lipid molecules, and ∼30,000 TIP3P water molecules, with a concentration of 0.15M Na^+^ and Cl^–^ ions. This approach resulted in a simulation system size of ∼120,000 atoms.

### MD Simulation

The simulation parameters was described by our pervious study (Cao et al., 2016). The Charmm36 all-atom force field (Vanommeslaeghe et al., 2010) was chosen for the MD simulation to obtain the equilibrated pentameric SARS-CoV-2 E protein model. The MD time step was set to 2 fs. Electrostatic interactions were described using the Particle Mesh Ewald (PME) algorithm (Darden et al., 1993) with a cut-off of 1.2 nm. The LINCS algorithm (Hess et al., 1997) was used to constrain the bond lengths. The pressure was maintained semi-isotropically at 1 bar in both the x and y directions using the Parrinello-Rahman barostat algorithm (Parrinello and Rahman, 1981), and the system temperature was maintained at 310 K using the Nose-Hoover thermostat (Evans and Holian, 1985). The time constants for both temperature and pressure were set to 1 ps. Then, 1000-ns (1-μs) MD simulations were performed for the pentameric E protein.

### Umbrella Sampling

The initial system used for umbrella sampling simulations was derived from the 1000-ns simulation of the pentameric SARS-CoV-2 E protein mentioned above, which sufficiently equilibrates the system. Single ions that maintain physiological activity (Na^+^, K^+^, Ca^2+^, Mg^2+^, and Cl^–^) or water molecules were placed at successive positions along the central pore axis by the GROMACS pull code. Energy minimization was performed before the simulation to optimize the water and ion positions. The reaction coordinate was defined from z +3 to −3 nm, with the mass center at *z* = 0 nm and a spacing of 0.1 nm between successive windows, resulting in 60 umbrella sampling simulation systems (see Supplementary Figure S1). The probe ions or water molecules were harmonically restrained by a force constant of 2000 kJ mol/nm^2^, consistent with the pore direction. Each window was used to perform a 30-ns total umbrella sampling simulation was performed using a time step of 3 fs. The initial 5 ns were used for system equilibration, and then the subsequent 25 ns were applied for analysis. The PMFs were computed using the weighted histogram analysis method (WHAM) (Rosenbergl, 1992), and the profile was generated using the GROMACS protocol ‘g_wham’ (Hub, 2015). A bootstrap analysis (*N* = 50) was performed to estimate the statistical error, the ‘-hist’ parameter was used to evaluate the quality of umbrella sampling (see Supplementary Figure S2) and the ‘-cycl’ parameter was used to normalize the values.

### Calculation the K^+^ Diffusion Coefficient

The diffusion coefficient was calculated using the method described by Shirvanyants et al. (2014). We restrained the E protein pentamer helix backbones and allowed the side chains, membrane, water and ions to freely move. Because the ion selectivity is related to the side chain of the inner pore (Gouaux and MacKinnon, 2005), the backbone restraint maintained the tertiary structure of the pentameric E protein pentamer, but the side chain flexibility in the inner pore was not altered. With K^+^ as the probe ion, the calculation system was used in the same manner as for umbrella sampling. The ion mean square displacement (MSD) was calculated along the z-axis of the pore. Twenty-five simulation systems was obtained, with each widow interval distance of K^+^ set as 0.24 nm. The umbrella restraint was used to maintain the position of the K^+^ ion on the x-y plane of the pore. The Einstein equation MSD = 2D(z)t was used to directly calculate the diffusion coefficient with the protocol g_msd. The umbrella restraint was disregarded for these analyses because the restraint force was negligible compared to the thermally induced RMS fluctuations.

### CE Simulations

Computational electrophysiology (CE) is a good tool for simulating the ion conduction ability of a channel under transmembrane voltage. We established a sandwich structure including three parts (membrane-protein-water), as described by Kutzner et al. (2011). Each water layer contained a different number of ions. Due to the imbalance of the ion distribution of the water layer, the ion gradient will produce transmembrane voltage. A specific transmembrane voltage was applied to the simulation system by adjusting the number of ions between the water layers. In the present study, we built the SARS-CoV-2 E protein sandwich system.

Each initial single-layer system was allowed to achieve equilibrium for 50 ns to stabilize the structure. Then the single-layer system was duplicated along the z direction. The size of the resulting system was approximately 11 nm × 11 nm × 22 nm and contained ∼250,000 atoms. The transmembrane voltage was set based on the different ion numbers of each part,

$$\Delta \,e=n\,u\,m\,{\left(1\right)}_{c\,l-}-n\,u\,m\,{\left(2\right)}_{c\,l-}=0\,e,4\,e,8\,e,12\,e$$

and the potential was calculated by implementing the Poisson equation ∇^2^⁡*U* = −ρ/ε in the GROMACS g_potential tool.

Three different systems were established to analyze the effect of the transmembrane voltage on the ions: NaCl, KCl, and CaCl_2_. Among them, the NaCl and KCl systems were simulated at transmembrane voltages of Δ*e* = 0, 4, 8 and 12e, while CaCl_2_ was simulated at Δ*e* = 8 and 12e. Each system was analyzed using a 50-ns simulation.

Afterward, six 20-ns simulations of each system were executed, and the total simulation time of each CE system was 680 ns. The details of each simulation are shown in Supplementary Tables S1 (initial system and umbrella sampling) and S2(CE simulation).

VMD software (Humphrey et al., 1996) was used to visualize the MD trajectories. All of the simulations were performed using the GROMACS 5.1 package (Abraham et al., 2015) on the Tianhe I Supercomputer in the National Supercomputer Center in Tianjin. The total simulation times were ∼4 μs.

## Results

### Sequence Alignment and Homology Modeling

**FIGURE 1:**
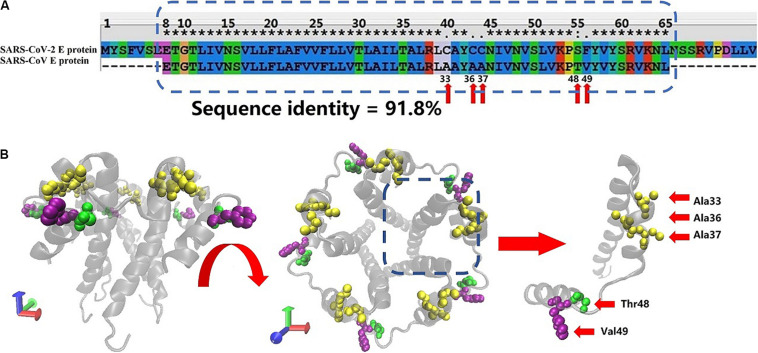
**(A)** Sequence alignment of the E proteins from SARS-CoV-2 and SARS-CoV. The SARS-CoV E protein sequence was obtained from PDB database (PDB ID: 5X29). The SARS-CoV-2 E protein sequence was obtained from NCBI. The sequences in the rectangular box were used for homology modeling. The red arrows mark the differences in amino acids between the SARS-CoV-2 and SARS-CoV E proteins. **(B)** Two views of the model of the SARS-CoV-2 E protein. The amino acids that differ between SARS-CoV and SARS-CoV-2 are marked with red arrows.

Figure 1A shows the sequence alignment of the E proteins from two human coronaviruses (SARS-CoV and SARS-CoV-2 sequence) generated using Clustal X software. The blue dotted rectangle in Figure 1B is the area of SARS-CoV-2 that corresponds to the crystal structure of the SARS-CoV E protein. The E protein amino acid sequences of these two coronaviruses are highly homologous, and the overall and transmembrane (TM) region similarity reached 91.8 and 100%, respectively.

### Evaluation of the Pentameric SARS-CoV-2 E Protein Homology Model

**FIGURE 2:**
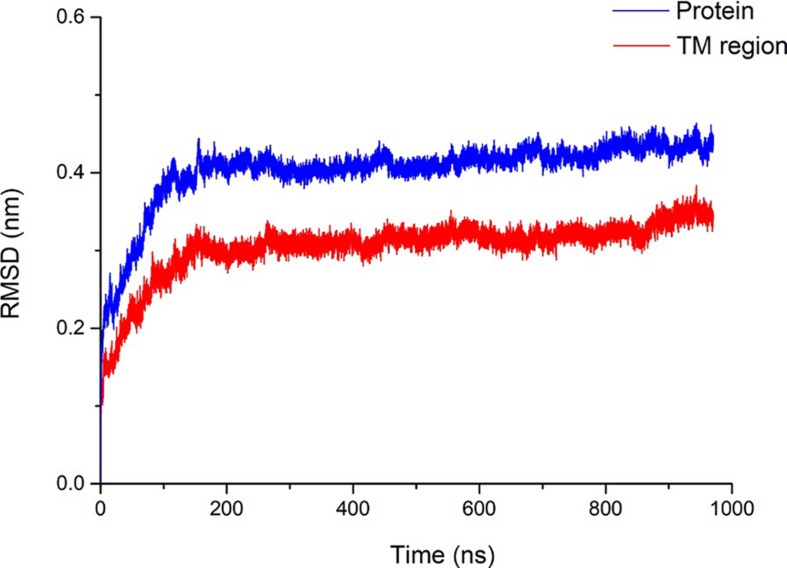
Structural stability of the SARS-CoV-2 E protein. The blue and red curves represent the root-mean-square deviation (RMSD) of the whole pentameric E protein and TM region, respectively.

The Rampage online program (Lovell et al., 2003) was used to evaluate the accuracy of the pentameric SARS-CoV-2 E protein model. More than 98.9% of the amino acids are within the acceptable range, suggesting that the SARS-CoV-2 E protein is similar to the NMR structure of the SARS E protein. Subsequently, a 1000-ns (1-μs) MD simulation was performed to evaluate the stability of pentameric E protein embedded in the membrane. The RMSD of the model is shown in Figure 2, and the red and blue curves represent the whole protein and TM region, respectively. In the first 200 ns, the RMSD continued to increase, indicating that the model needed a longer optimization period than other ion channels to reach equilibrium of the pentameric structure. During the last 800 ns, the curves plateaued. The RMSD of the whole protein and the TM region converged at ∼0.4 and ∼0.3 nm, respectively. A 0.1-nm difference was observed between the whole protein and TM region. These findings are consistent with other ion channel data showing that the TM region has a higher stability than other parts of the membrane protein (Khalili-Araghi et al., 2009).

### Water and Ion Permeability in the Dewetted Pore

The permeability of the pentameric SARS-CoV-2 E protein channel is a very important parameter to measure for an understanding of the replication ability of viruses in cells and how they are secreted into the extracellular medium (Ruch and Machamer, 2012). A profile of the free energy of a single ion or water as a function of its position along the pore axis is measured by calculating the PMF. In this simulation, the ions or water molecules were restrained to a continuous position along the z-axis and moved freely in the xy plane of the pore. Moreover, other parts of the system (proteins, ions, water molecules, and lipids) moved freely and reached equilibrium. The pore was maintained in the dewetted state during the umbrella sampling simulation.

**FIGURE 3:**
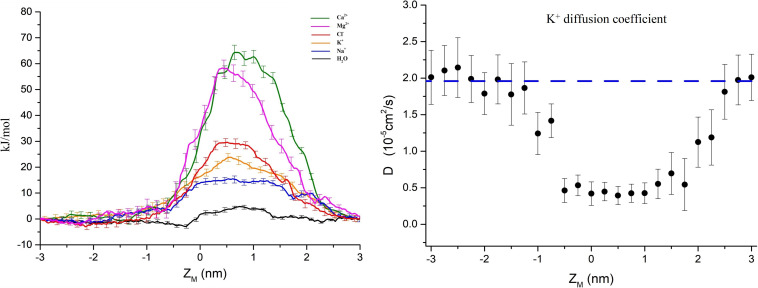
**(A)** PMF profiles of single ions (Ca^2+^, Mg^2+^, Cl^–^, K^+^, Na^+^, and H_2_O represented by green, magenta, red, yellow, blue, and black curves, respectively) as a function of position along the pentameric SARS-CoV-2 E protein pore axis. **(B**) The K^+^ diffusion coefficient profile. The dashed line represents the experimental value of a single K^+^ diffusion coefficient in water, D = 2.0 × 10^– 5^ cm^2^/s. Error bars were estimated by bootstrapping and are the same color as the corresponding curves. Z_*M*_ = 0 for the center of mass of the E protein pentamer.

The PMFs of important physiological ions (Mg^2+^, Ca^2+^, Cl^–^, K^+^, and Na^+^) and the water molecules as a function of their position along the pore z-axis were calculated separately. Figure 3A shows the PMFs of ions and water molecules permeating through the pentameric SARS-CoV-2 E protein pore. Z_*M*_ is defined as the axial direction along the pore, which ranges from −3 ∼ 3 nm (the length of umbrella sampling is 6 nm in total). From the PMF curve, the free energy barriers of monovalent and divalent ions were significantly different. The maximum free energy barriers of the two divalent ions Ca^2+^ and Mg^2+^ were 68 and 60 kJ/mol, respectively. In contrast, the PMFs of the monovalent ions Na^+^ and K^+^ were ∼12 and ∼20 kJ/mol, respectively, while the Cl^–^ free energy barrier was ∼30 kJ/mol, a value that was significantly smaller than the PMFs of Ca^2+^ and Mg^2+^. From a free energy perspective, the pentameric SARS-CoV-2 E protein is almost impermeable to divalent ions. This finding confirms the previous hypothesis that the SARS-CoV and MERS-CoV E protein channels are monovalent cation channels (Wilson et al., 2004; Surya et al., 2015). The maximum free energy barrier in descending order is as follows: Na^+^ < K^+^ < Cl^–^ < Mg^2+^ < Ca^2+^. Additionally, the barrier of water molecules is only ∼5 k/mol, which is similar to the free energy threshold of free diffusion in liquid. Interestingly, no obvious peak was identified in the PMF curve for Na^+^ ions and water molecules. Based on these results, the pentameric E protein pores are not permeable to both monovalent and divalent ions in the dewetted state. However, the difference in the free energy barrier of the monovalent cations is large, suggesting that the channel is selective for ions.

We calculated the diffusion coefficient (D) using a single K^+^ ion as a probe to explore the permeability of the channel. By fitting the K^+^ MSD with the Einstein equation (MSD = 2Dt), we obtained the diffusion coefficient. As shown in Figure 3B, the average diffusion coefficient of K^+^ outside the pore was ∼2.0 × 10^–5^ ± 0.4 cm^2^/s, consistent with the experimental data obtained for K^+^ in the liquid environment (Hille, 2001). The D decreases by approximately 70% when the K^+^ probe is in the channel pore. The D curve of the internal channel pore has a flat bottom and is similar to the PMF. The mid-value of D = 0.35 × 10^–5^ ± 0.2 cm^2^/s indicates the presence of a large obstacle for probe diffusion in the inner pore, showing a similar value to other narrow hydrophobic pores (Roux et al., 2004; Trick et al., 2016).

Next, we used the solubility-diffusion equation (Eq. 1) (Marrink and Berendsen, 1996; Sun et al., 2018) to evaluate the permeability coefficient (P) between two different ions.

(1) $$\frac{1}{P}=\int_{z\,1}^{z\,2}\frac{\exp\left(\frac{P\,M\,F\,\left(z\right)}{k_{b}\,T}\right)}{D\,\left(z\right)}\,dz$$

In Eq. 1, PMF(z) and D(z) are functions of PMF and D along the z-axis, z1 denotes a position on one side of the pore and z2 denotes a position on the other side. _*k_b*_ is the Boltzmann constant, and T is the simulation temperature. The part of the PMF in the TM region is approximately the same as the graph of the quadratic function, and thus the PMF(Z) uses a quadratic function fit. The linear fit of the corresponding D gives the function D(z). The ratios of the *P*-values for Na^+^, K^+^, and Cl^–^ in the maximum free energy barrier region were simply calculated: P_*Na*__+_/P_*K*__+_ = 22.42, P_*K*__+_/P_*Cl–*_ = 5.82, and P_*H2O*_/P_*Na*__+_ = 402.57.

### Computational Electrophysiology

**FIGURE 4:**
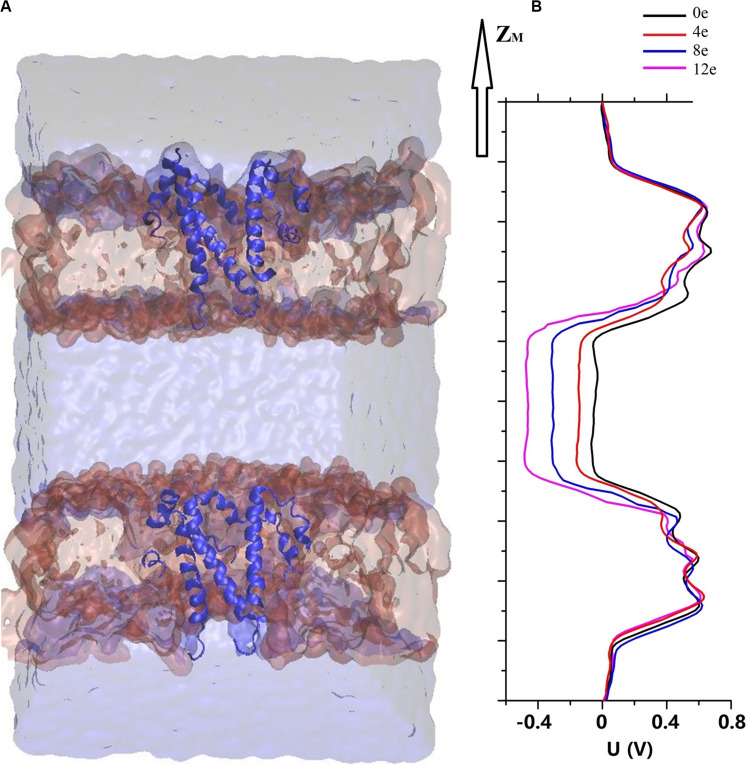
**(A)** A sandwich simulation system of SARS-CoV-2 in the presence of 0.15M NaCl containing the pentameric SARS-CoV-2 E protein, cell membrane, ions, and water molecules. **(B)** The electrostatic potential ΔU along the z-axis arising from imbalances (ΔQ) between the elementary charges of 0 and 12 (colored curve).

According to previous electrochemical experiments, the transmembrane voltage exerts an important effect on the pore permeability of the E proteins in both SARS-CoV and MERS-CoV (Wilson et al., 2004; Surya et al., 2015). In addition, viroporins and, similarly, nanopores are sensitive to the transmembrane voltage, causing electrowetting of the pores and changing the tension of the hydrophobic surface inside the pores, which is sufficient to functionally open the channels (Bratko et al., 2007; Catterall, 2010; Nieva et al., 2012; Trick et al., 2017). We used the computational electrophysiology first proposed by Kutzner et al. (2011). This approach expands the membrane protein structure into a “sandwich” and simulates the transmembrane voltage by adjusting the number of ions in different layers. It directly simulates and analyzes the ion penetration in the presence of transmembrane voltage at an atomic resolution. The sandwich structure of the pentameric SARS-CoV-2 E protein is presented in Figure 4A. Four models were established to simulate the charge differences between the two sides of the channel (ΔQ = 12e, 8e, 4e, and 0e). Using the method described above, the transmembrane voltage was calculated as ΔU = ∼0.45, 0.3, 0.15, and 0 V, respectively (Figure 4B).

### Water Behavior Under a Transmembrane Voltage

According to Trick et al. (2016), the behavior of water molecules in pores is a reasonable method to study the ion permeability of channels. Water influx into hydrophobic pores may help ions overcome the free energy barrier of hydrophobic surfaces (Trick et al., 2014; Yamashita et al., 2017). Overall, we evaluated the hydration properties of the pentameric E protein pore according to the statistical analysis of the number of water molecules in the inner pore.

**FIGURE 5:**
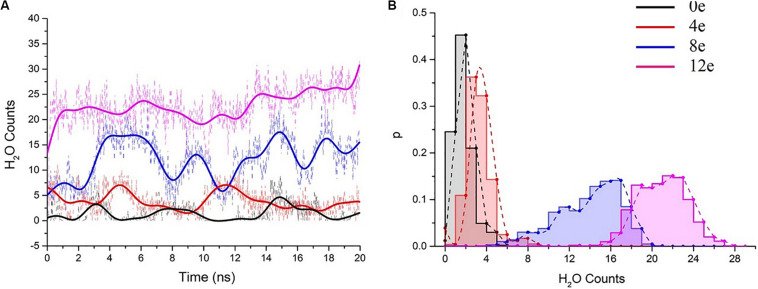
**(A)** The number of pore water molecules as a function of time in the presence of different transmembrane voltages. The scatter plot presents the absolute numbers of water molecules, and the colored curves present the number of water molecules after smoothing (*n* = 25) using the fast Fourier transformation (FFT) method. **(B)** Normalized probability histogram of the number of water molecules in the TM region during 20-ns simulations. The different colors represent different models: 0e (blue), 4e (red), 8e (blue), and 12e (magenta).

We performed six 20-ns simulations for each transmembrane voltage to accurately assess the behavior of water molecules in the pentameric SARS-CoV-2 E protein pores, and the total simulation time reached ∼0.7 μs. Subsequently, the water molecules in the system at different transmembrane voltages were counted (the statistical analysis of the changes in the molecule counts are shown in Supplementary Figure S3). We extracted representative water molecule diagrams for each voltage, as shown in Figure 5A. When ΔQ = 0e, the time for the channel to remain wet is ∼3 ns. ΔQ = 4e is slightly longer (∼5 ns). The absolute number of water molecules in the pore is small, with the average value ranging from 0 to 5 (Figure 5B). Although the pores are functionally open for water permeation, due to the small stream of water, they may not be able to maintain sufficient electrical wetting, resulting in ions that are unable to permeate the pore unimpeded. We speculate that at the maximum physiological voltage (∼0.2 V), the pentameric SARS-CoV-2 E protein pore only permeates a few water molecules.

The number of water molecules increased significantly when ΔQ = 8e or 12e (ΔU = ∼0.3 and 0.45 V, respectively), and the water distributions were 4–20 (8e) and 15–27 (12e), respectively (for the process of water permeation, see Supplementary Movie S1). The pores remained in a wet state.

### Behavior of Monovalent and Divalent Ions Under a Transmembrane Voltage

Additionally, we analyzed the trajectories of the ΔQ = 8e and 12e (ΔU = 0.3 and 0.45 V) double layer system. A few water molecules were able to be co-transported with Na^+^ and K^+^ during the high voltage simulations (see Supplementary Movies S2, S3). Interestingly, as the voltage increased (from 0.3 to 0.45 V), the permeability of water molecules also increased synchronously, showing a periodic change regardless of the magnitude of the transmembrane voltage (even if ΔU = 0). We further simulated the CaCl_2_ system at 0.45 V and observed that Ca^2+^ permeated through the pore (see Supplementary Movie S4). The maximum ΔU = 0.45 V is not sufficient to cause electrical breakdown of the lipid bilayer (Benz and Zimmermann, 1980), indicating that the SARS-CoV-2 E protein channel is activated by transmembrane voltage.

The behaviors of water and ions in the presence of different transmembrane voltages strongly suggests that the pentameric SARS-CoV-2 E protein channel has obvious wetting/dewetting transitions and the typical characteristics of voltage-dependent hydrophobic channels.

### Pore Conformation Analysis

**FIGURE 6:**
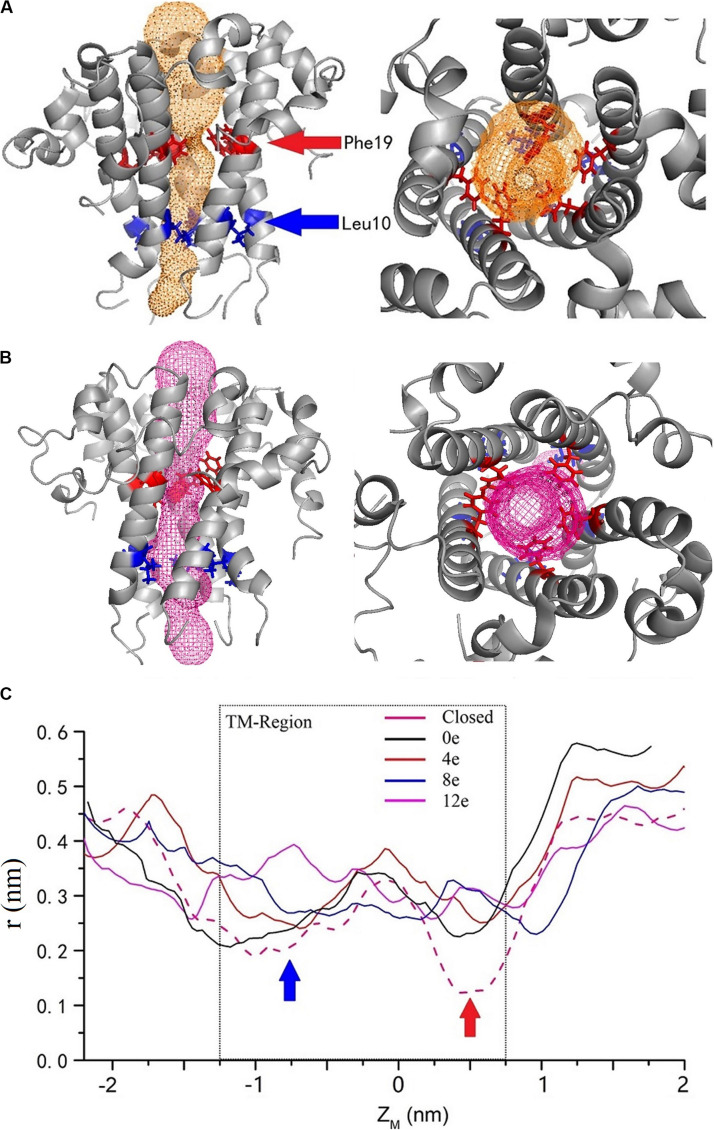
The channel structures in the closed **(A)** and completely open **(B)** state. The red and blue arrows indicate the two residues with the largest changes in radius at different voltages. **(C)** The variation in the inner pore radius. The z axis shows the distance from the center of mass. The solid black, red, blue, and magenta curves represent ΔQ = 0e, 4e, 8e, and 12e, respectively, and the pink dashed curve represents the closed state. The calculated channel measured using CAVER 3.01 (Chovancova et al., 2012).

By calculating the channel radius at different voltages [ΔU = ∼0.45, 0.3, 0.15, 0, and 0 V (closed state)] (Figure 6B), two positions in the TM region of the pentameric E protein channel in the closed state (ΔU = 0 V) exhibited the minimum radius, *r* = 0.2 nm (Z_*M*_ = −0.8 nm) and 0.15 nm (Z_*M*_ = −0.5 nm), corresponding to Leu10 and Phe19 (blue and red arrows, respectively). Phe19 and Leu10 are located at the top and bottom of the pore, respectively. The two positions represent a candidate gated area of the channel. As shown in Figure 6A, these two locations represent obvious “bottlenecks,” which are not large enough for water to permeate. This result was also confirmed by calculating the channel radius at different transmembrane voltages. The radius of the pore obviously increased with transmembrane voltage at these two locations. In the open state (ΔU = 0 V), the pore radius increased to 2 and 2.2 nm. When the transmembrane voltage increased to ΔU = ∼0.15 and 0.3 V, the radius of the pore at the Leu10 position was ∼0.23 and 0.28 nm, and the radius of the pore at Phe19 was ∼0.25 and 0.33 nm, respectively. The voltage exerted a greater effect on Phe19 than on Leu10. However, when ΔU = 0.45 V, the radius of the pore was still ∼ 0.33 nm at Phe19 but had expanded to 0.4 nm at Leu10, indicating that a large transmembrane voltage may make Leu10 excessively available, which may disrupt the unidirectional permeation. The narrowest locations of the inner pore are consistent with the free energy barrier. Apparently, the radius of the inner pore showed that Leu10 and Phe19 of the SARS-CoV-2 E protein are candidate hydrophobic gates.

## Discussion

COVID-19 is an unprecedented threat to humanity. The replication mechanism of the virus in the human body is particularly important to understand the infection. Currently, research on the pathogenic mechanism and infection route of COVID-19 mainly focuses on the S protein (Shereen et al., 2020; Smith and Smith, 2020), but research on the E protein is lacking. Interestingly, the E protein can protect the spike protein to promote virus infection (Westerbeck and Machamer, 2019). The E protein pentamers from different corona virus subtypes (SARS-CoV and MERS-CoV) may be viroporins, which play an important role in the virus life cycle (Gonzalez and Carrasco, 2003). Deletion of the gene encoding the E protein will substantially reduce the replication and pathogenicity of coronaviruses. Moreover, the channel activity of the pentameric E protein is necessary for inflammasome activation and is the determinant of coronavirus virulence (Jimenez-Guardeno et al., 2015). Currently, many atypical symptoms are present in patients with COVID-19, such as gastrointestinal and neurological symptoms, including abdominal pain with diarrhea and the disruption of smell and taste, respectively (Ai et al., 2020; Mao et al., 2020). These symptoms may also be closely related to the abnormal ion permeability caused by the E protein viroporin (Ortego et al., 2007; Taruno et al., 2013).

This study explored the permeability mechanism of the pentameric SARS-CoV-2 E protein. We used the NMR structure of the SARS-CoV E protein pentamer to build the model of the pentameric SARS-CoV-2 E protein using homology modeling. A combination of MD simulations, PMF, diffusion coefficient calculations, computational electrophysiology analyses, and channel pore geometry analyses was used. The results were mutually corroborated and characterized the TM dynamics of the pentameric SARS-CoV-2 E protein channel. The channel allowed free flow of water, but the cations (Na^+^, K^+^, and Ca^2+^) were only transported in the wetted state, suggesting that this protein is a cation-selective voltage-dependent hydrophobic channel. Two amino acids, Leu10 and Phe19, are candidate hydrophobic gates.

**FIGURE 7:**
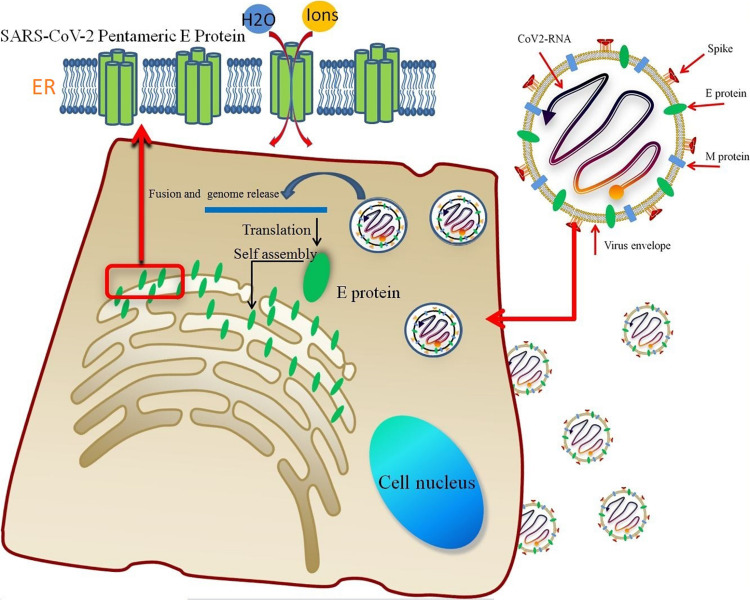
The lipid envelope encloses the virus and facilitates the entry of the SARS-CoV-2 E protein into the host cell. The E protein is translated in the ER and accumulates in the Golgi. Then, the E protein monomer self-assembles into an oligomer that functions as an ion channel.

The pentameric SARS-CoV-2 E protein has been considered an ion channel, and studies aiming to probe its properties are very worthwhile. It is translated into monomers in host cells and self-assembled in the ER (Figure 7). A significant similarity in the TM region sequences (reaching 91.8%) and high gene homology between the SARS-CoV and SARS-CoV-2 E proteins has been observed; five different residues are all located at the region of interaction between the protein and membrane, while the sequences of the TM region are completely identical. The highly conserved TM region also suggests similar functions for E proteins from different coronavirus subtypes. Based on the RMSD of the E protein pentamer during the 1-μs MD simulation, the structure exhibits a certain amount of flexibility, showing that its geometric deformation increases the permeability of the channel to ions and water molecules. As shown in Figure 2, the TM region of the SARS-CoV-2 E protein remains relatively stable during the simulation, indicating the stability of the pentamer in the membrane environment, consistent with previous studies of SARS-CoV and MERS-CoV.

The free energy calculation of the ions permeating through the pentameric SARS-CoV-2 E protein channel strongly suggests that the pore displays selective permeability for monovalent ions. The maximum free energy barriers of Na^+^, K^+^, and Cl^–^ are 15, 20, and 30 kJ/mol, respectively. The comparison of the *P*-values for Na^+^, K^+^, and Cl^–^ shows that the P of Na^+^ is 22 times greater than K^+^, while the P of K^+^ is 6 times greater than Cl^–^. Although the computational permeability coefficient is two times higher or lower than the value observed experimentally for SARS-CoV, it still shows a very large gap in the permeation between ions and water molecules. Based on our simulation, SARS-CoV-2 exhibits a difference in ionic selectivity. As shown in Figures 1A,B, the five residues that differ between SARS-CoV and SARS-CoV-2 are located outside of the pore (A33C, A36C, A37C, T48S, and V49F, respectively, see Supplementary Figure S4). The residues at positions 33, 36, and 37 changed from the non-polar alanine to polar cysteine (Ala to Cys), allowing water molecules to more easily aggregate around the entrance of the channel, increasing the probability of water molecule permeation, and maintaining the pore in a wetted state allowed water and ions to more easily permeate through the channel. The residue at position 49 is replaced with phenylalanine (Val to Phe), and the benzene ring side chain binds tightly with the membrane, increasing the stability of the E protein pentamer in the membrane. Thus, compared with the SARS-CoV E protein, the SARS-CoV-2 E protein requires a higher transmembrane voltage to open the channels, which is consistent with our computational electrophysiology result (ΔUcov:ΔUcov2 = 0.1:0.15 V).

Additionally, no obvious peak for the PMF was detected when Na^+^ and water molecules permeated through the channel, supporting the hypothesis that Na^+^ and water molecules have less resistance to penetration of the inner pore. This finding further confirmed the selectivity for Na^+^. However, the PMF indicates that all ions are unable to overcome this very large free energy barrier and spontaneously permeate through the dewetted pore. Interestingly, Nieto-Torres et al. (2015) revealed that the SARS-CoV E protein is also a calcium transporter. In our simulation, the very high PMF of Ca^2+^ (∼70 kJ/mol) in the dewetted state suggests that Ca^2+^ is unlikely to permeate the channel. However, the CE simulation revealed that the 0.45 V transmembrane voltage activates Ca^2+^ permeation through the channel. Several explanations for this result are proposed below. (1) The geometric size of the inner pore does not provide sufficient space for Ca^2+^ in the dewetted state, causing the high free energy barrier (Figure 6, the radius is ∼0.1 nm). (2) Ca^2+^ possesses the highest asymmetrical distribution between the ER-Golgi lumen and the cytoplasm (Zhou et al., 2009); the gradient of calcium creates a transmembrane voltage that completely opens the pore and promotes the influx of water molecules caused Ca^2+^ permeating through the pore. (3) The simulation of the bilayer (PE:PS:PC = 3:1:1) with a mixed charge altered the ion permeability and selectivity of the E protein channel, as described by Verdiá-Báguena et al. (2012). Further simulations to clarify the properties of the SARS-CoV-2 channel and the effects of lipid charges are in progress.

An important effect of the transmembrane voltage on viroporins has been observed in many viruses, including HIV1 Vpu oligomers and the hepatitis C virus (HCV) p7 protein, etc. (Schubert et al., 1996; Clarke et al., 2006; Mehnert et al., 2008). Although it has been observed in many experiments, this mechanism has not been elucidated. Because water molecules play an important role in hydrophobic ion channels, their behavior at the inner pores under different voltages (ΔU = ∼0.45, 0.3, 0.15, and 0 V) was the focus of this simulation. Computational electrophysiology enabled the determination of a conducive conformation for ions. The channel wetting/dewetting transitions appeared at different transmembrane voltages, and the radius of the pore increased. Meanwhile, the capacity to contain water molecules also increased. Further analysis of the trajectories revealed that the monovalent cations (Na^+^, K^+^, and Ca^2+^) will permeate through the pore with water molecules when the transmembrane voltage is > 0.3 V.

Computational electrophysiology also explained that the transmembrane voltage led to an easier wetting transition for the channel. The range of voltages required to maintain the channel in the open state was 0.15 and 0.45 V. Na^+^, K^+^, and Ca^2+^ might be co-transported during water permeation, which is very similar to the many other hydrophobic channels (McCormack et al., 1991; Powell et al., 2011; Smirnov et al., 2011; Aryal et al., 2015). Intriguingly, we did not observe Cl^–^ conduction. In addition, the change in the pore radius at different voltages highlights two important sites with the greatest change in the geometric radius, namely, Leu10 and Phe19. Phe19 contains a benzene ring group at the hydrophobic surface of the inner pore as a hydrophobic gate. The isopropyl group of Leu10 at the bottom of the pore prevents reverse transport (Figure 6A). Asn15Val and Val25Phe mutations will make the channel dysfunctional, potentially due to the additional side chain groups located in the pores containing these mutations (particularly the benzene ring in Val25Phe), thus (1) decreasing the radius of the pores and (2) increasing the hydrophobicity of the pores. In summary, these mutations may increase the free energy barrier, negatively affecting the wetting transition and causing the channel to be functionally closed.

Therefore, the pentameric SARS-CoV-2 E protein is a voltage-dependent hydrophobic channel. We propose that the E protein may play an essential role in the viral infection and replication processes through the mechanisms listed below. (1) The abnormal ion concentration gradient causes a fluctuation in the transmembrane voltage, resulting in abnormal H^+^ flow and altering the intracellular pH (the virus viroporins allow H^+^ ions to permeate) (Wozniak et al., 2010). (2) The SARS-CoV-2 E protein viroporin regulates the transmembrane voltage to maintain the microenvironment within a range that is suitable for ion and water permeation, providing a suitable microenvironment for virus replication. (3) Selective permeation is caused by the transmembrane voltage, creating feedback regulation and maintaining an intracellular microenvironment through the protonation and deprotonation of the titratable residues present in the E protein channel pore, which are suitable for viral growth. (4) The disintegration of the ion equilibrium of the intracellular area affects the charges coming from the cell through ion channels in the cell membrane, changing the pH and enabling the virus to fuse with the cell membrane. (5) The pH variation has a certain signal transduction function. These infection mechanisms have been observed in many viruses, such as coronaviruses, the hepatitis C virus, influenza viruses, and HIV (Premkumar et al., 2004; Nieva et al., 2012; Verdiá-Báguena et al., 2012, 2013).

However, in our simulation, we used a mixed lipid membrane model composed of PE, PS and PC consistent with the experimental method, which is still insufficient to reflect the real lipid environment (including many neutral and charged lipid membranes, such as cholesterol, sphingolipids, PC, PI, PS, etc.). Different membrane components affect the permeability of the E protein pentamer (Verdiá-Báguena et al., 2012, 2013). In particular, cholesterol is very important for viral membrane fusion, vesicular transport of the E protein, and membrane protein self-assembly (Tseng et al., 2010; Meher et al., 2019). In conclusion, the lipid membrane models must be carefully analyzed and interpreted in future studies; in particular, cholesterol must be incorporated into these models to clarify its role in determining the permeability of the pentameric E protein.

## Conclusion

Although the crystal structure of the pentameric SARS-CoV-2 E protein is not yet available, the channel conformation was established by homology modeling of the pentameric SARS-CoV E protein. We characterized and estimated the stability, free energy barrier, voltage dependence, and geometric properties of the channel using MD simulations. Our simulation results reveal that the pentameric SARS-CoV-2 E protein is a voltage-dependent hydrophobic channel with monovalent cation selectivity. Water molecules and monovalent cations spontaneously penetrate through the channel under a transmembrane voltage. Differences in the penetrability of E protein channels may be responsible for the infectivity, inflammatory response and incubation period of SARS-CoV-2, which differ from SARS-CoV and MERS-CoV. Furthermore, Leu10 and Phe19 are hydrophobic gates that regulate ion permeability. In summary, the function of E protein ion channels plays a key role in viral replication, release and cytotoxicity. Our study reveals the properties of the pentameric SARS-CoV-2 E protein, providing theoretical support for understanding the abnormal symptoms and aiding in the development of vaccines and treatments for the cytokine storms caused by SARS-CoV-2 and other coronaviruses.

## Data Availability Statement

The datasets presented in this study can be found in online repositories. The names of the repository/repositories and accession number(s) can be found in the article/ Supplementary Material.

## Author Contributions

YC, IL, RY, and BX contributed to conception and design of the study. YC, JS, RZ, and WZ performed the simulation and analysis. YC, RY, RZ, and XM contributed to the analysis and interpretation of data and drafting. IL and BX revised the manuscript. YC, WW, and BX provided the funding. All authors contributed to the article and approved the submitted version.

## Conflict of Interest

The authors declare that the research was conducted in the absence of any commercial or financial relationships that could be construed as a potential conflict of interest.
