# Supplementary material for: Computational Study of the Ion and Water Permeation and Transport Mechanisms of the SARS-CoV-2 Pentameric E Protein Channel
Source: Front Mol Biosci. 2020 Sep 23;7:565797. doi: 10.3389/fmolb.2020.565797 (PMC7538787; doi:10.3389/fmolb.2020.565797)
Supplement: Supplementary file 1 [file Table_1.DOCX]

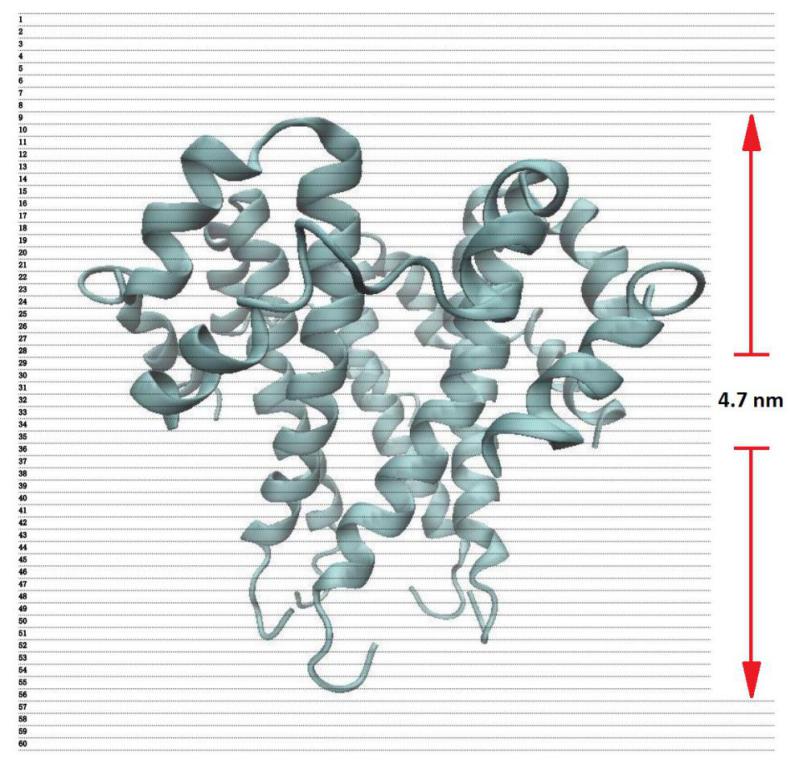


Supplementary Figure S1. The schematic diagram of umbrella sampling. The total number of widows are 60 with spacing 0.1 nm between successive windows. The length of E-protein is about 4.7 nm.


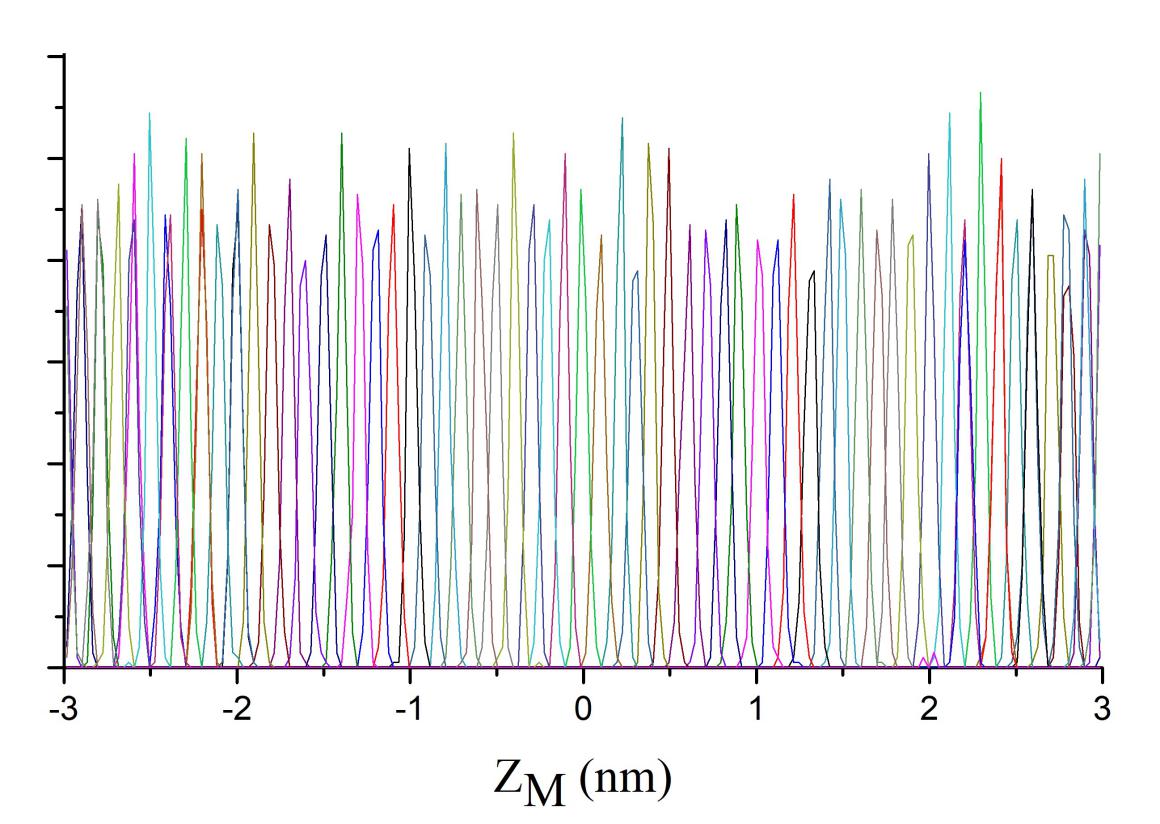


Supplementary Figure 2. The hist plot of umbrella sampling. All of the windows centered on this region of the reaction coordinate.


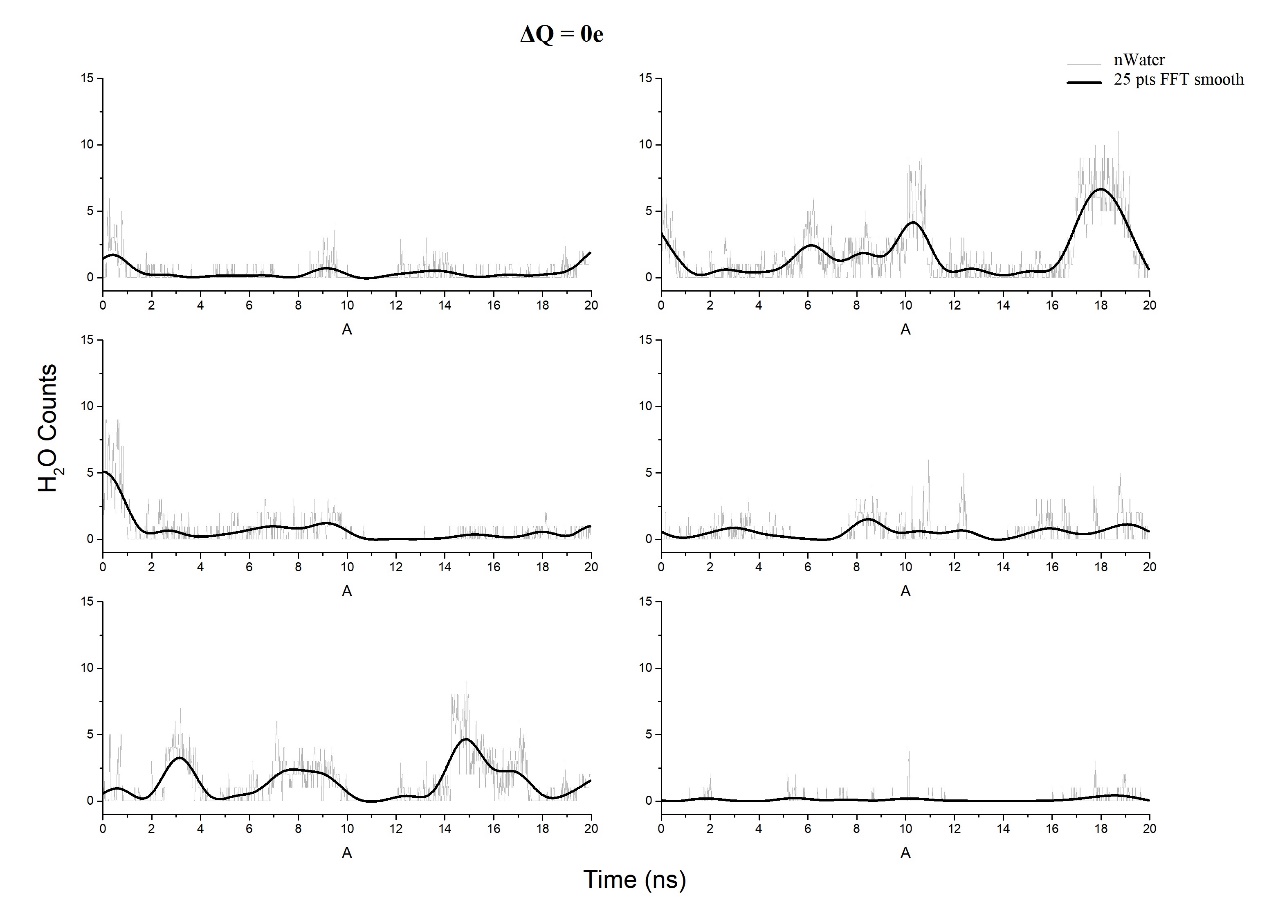

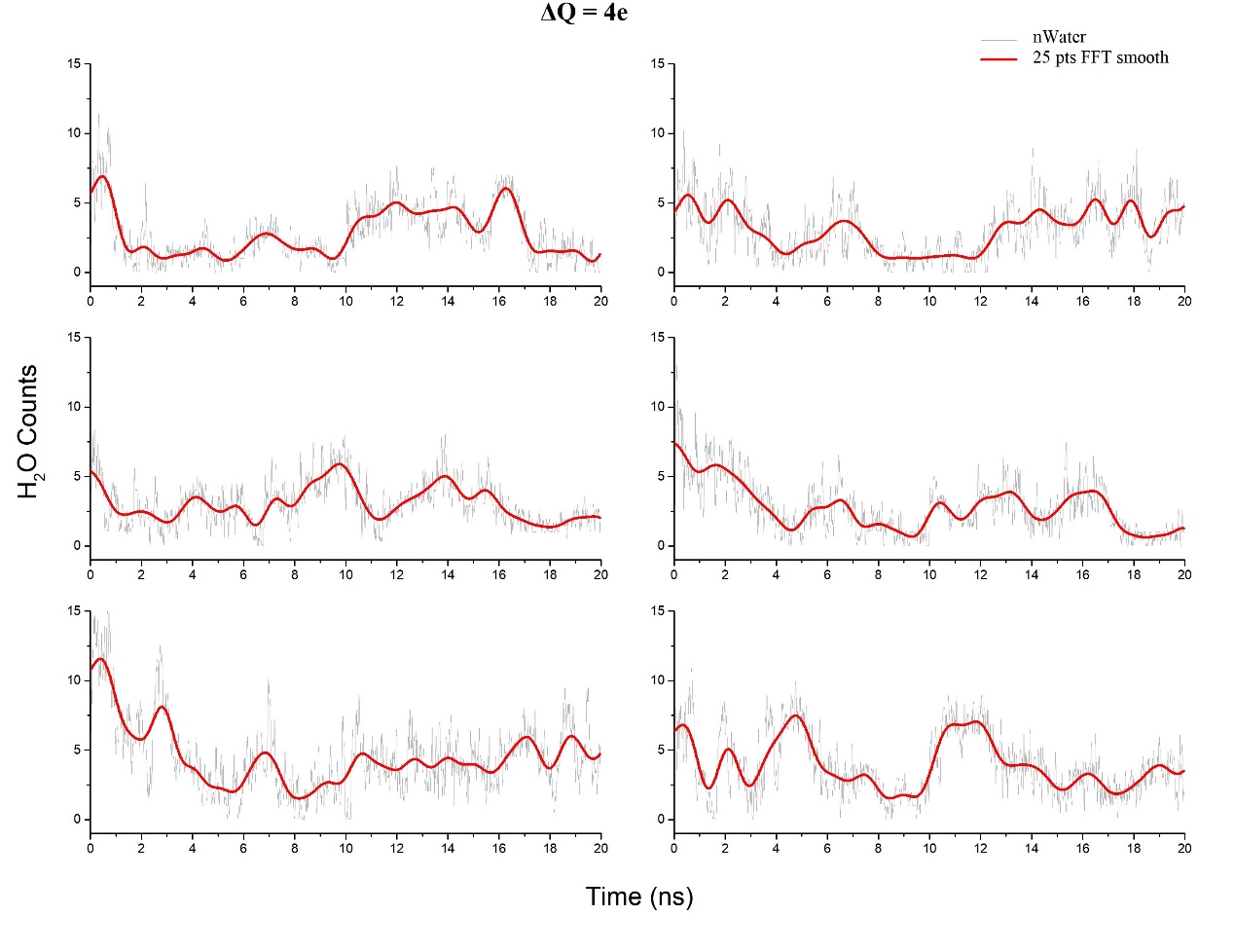

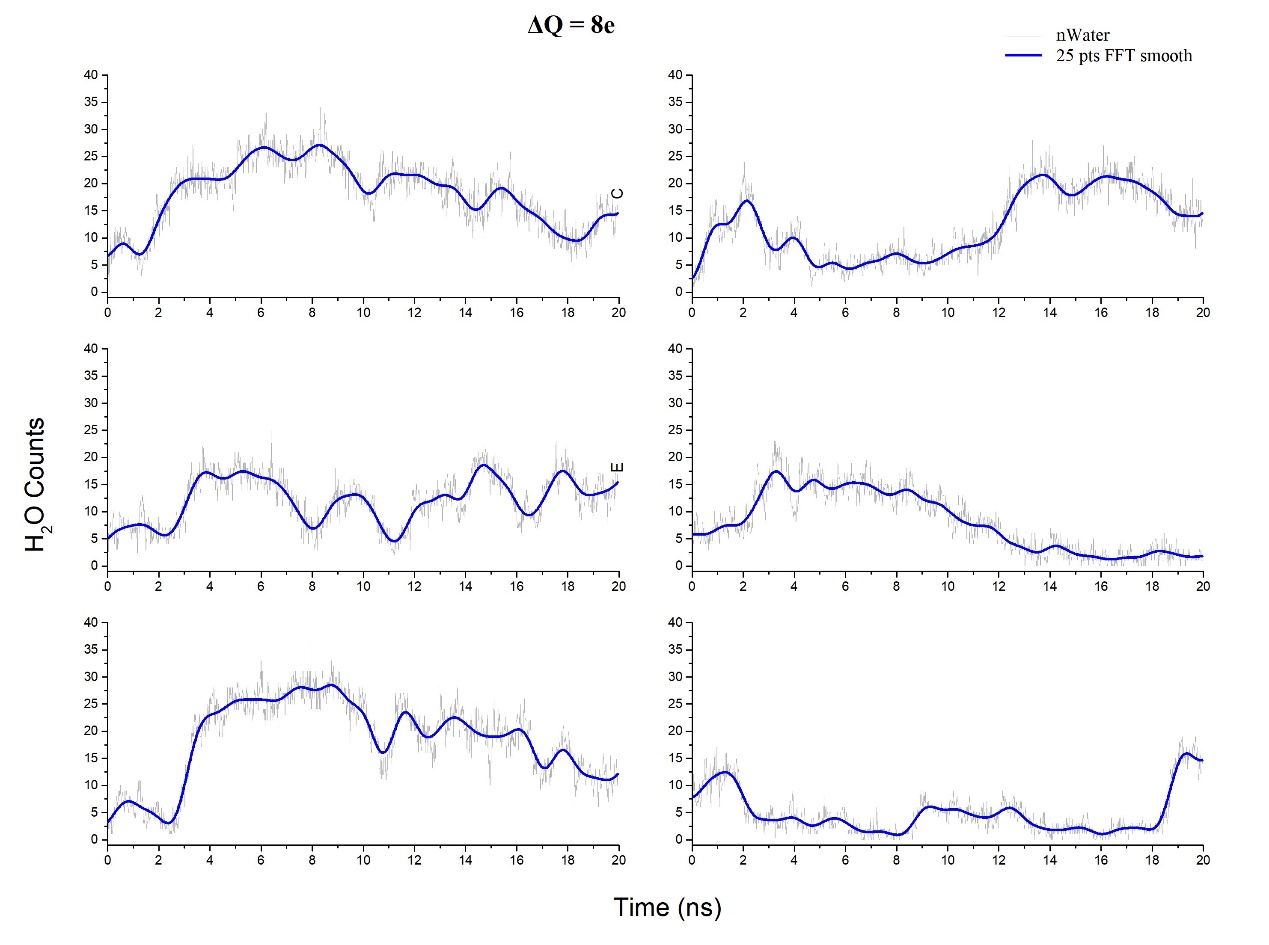

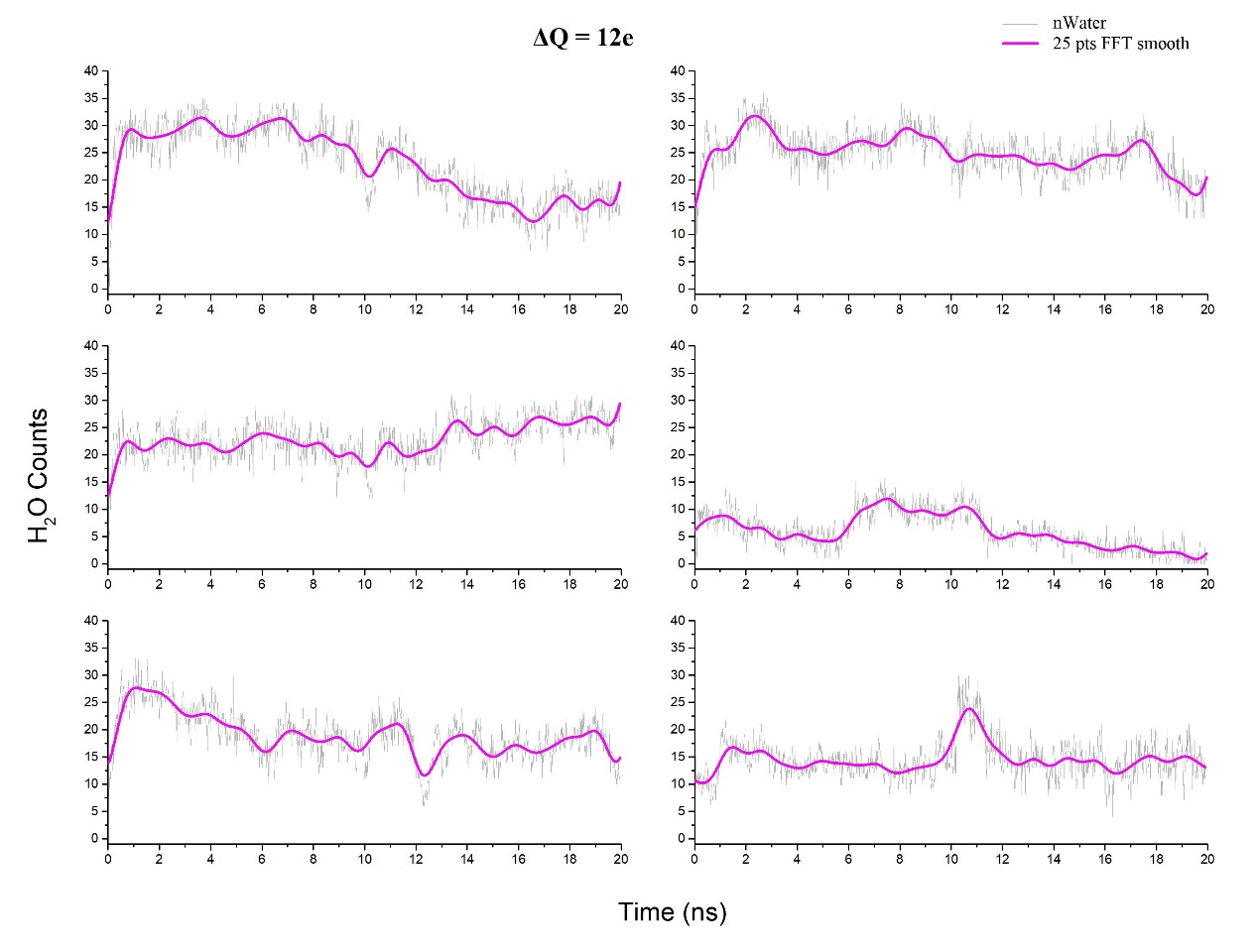


Supplementary Figure S2. statistical data of the changes in the molecule counts of ΔQ= 0e, 4e, 8e, 12e respectively. The scatter represents the absolute counts of water molecules, and the color curve represents after smoothing.


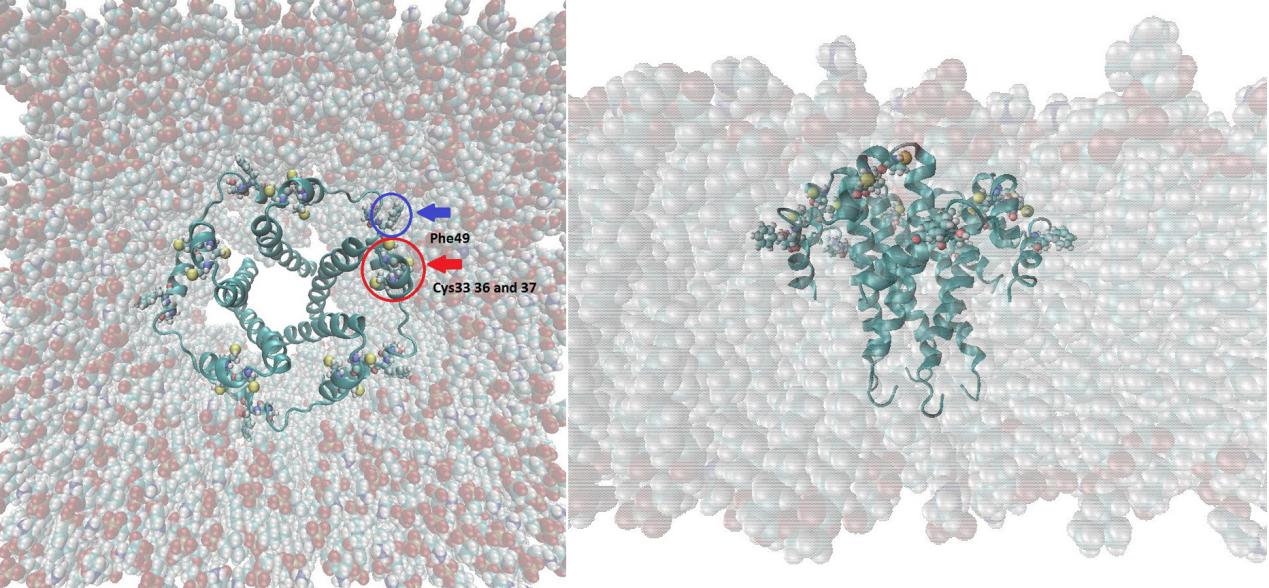


Supplementary Figure S3. The E-protein pentamer embeded in membrane. The red and blue arrow represent the Cys33, 36, 37 and Phe49 respectively.
