# Supplementary material for: Computational Study of the Ion and Water Permeation and Transport Mechanisms of the SARS-CoV-2 Pentameric E Protein Channel
Source: Front Mol Biosci. 2020 Sep 23;7:565797. doi: 10.3389/fmolb.2020.565797 (PMC7538787; doi:10.3389/fmolb.2020.565797)
Supplement: Supplementary file 2 [file Table_2.DOCX]

Table1, the single bilayer system simulation setup details statistical table

|  | bilayer setup | system atoms | simulation time (ns) | Number of ions (or H_2_O) | Simulation time for each repeats（ns） | Totally time (ns) |
| --- | --- | --- | --- | --- | --- | --- |
| System simulation | single | ~120,000 | 1000 |  | 1000 | 1,000 |
| Umbrella sampling | single | ~120,000 | 60 per ions (H_2_O) | 6 | 30 | 10,800 |

Table2, CE simulation setup details statistical table

| Ions (H2O) system | bilayer setup | system atoms | number of simulation repeats | Number of voltage | Total Repeat times | The simulation time for each repeat (ns) | Pre- equilibrium time (ns) | Totally time (ns) |
| --- | --- | --- | --- | --- | --- | --- | --- | --- |
| H_2_O | double | ~240,000 | 6 per voltage | 4 | 24 | 20 | 50 | 530 |
| NaCl | double | ~240,000 | 1 per voltage | 4 | 4 | 50 | 50 | 250 |
| KCl | double | ~240,000 | 1 per voltage | 4 | 4 | 50 | 50 | 250 |
| CaCl_2_ | double | ~240,000 | 1 per voltage | 2 | 2 | 50 | 50 | 150 |
