# Supplementary material for: Computational Study of the Ion and Water Permeation and Transport Mechanisms of the SARS-CoV-2 Pentameric E Protein Channel
Source: Front Mol Biosci. 2020 Sep 23;7:565797. doi: 10.3389/fmolb.2020.565797 (PMC7538787; doi:10.3389/fmolb.2020.565797)
Supplement: Supplementary file 3 [file Table_3.DOCX]

@ title "Mean Square Displacement"

@ xaxis label "Time (ps)"

@ yaxis label "MSD (nm\S2\N)"

@TYPE xy

# MSD gathered over 4200 ps with 71 restarts

# Diffusion constants fitted from time 420 to 3780 ps

# D[ POT] = 0.3919 (+/- 0.1588) (1e-5 cm^2/s)

0 0

15 0.0395631

30 0.0644386

45 0.103857

60 0.122048

75 0.144895

90 0.172253

105 0.17779

120 0.177688

135 0.181534

150 0.213191

165 0.230522

180 0.222355

195 0.224891

210 0.234195

225 0.271602

240 0.262519

255 0.292778

270 0.288171

285 0.320864

300 0.304391

315 0.358964

330 0.369295

345 0.400206

360 0.3746

375 0.438866

390 0.43009

405 0.466551

420 0.427761

435 0.482219

450 0.501127

465 0.533352

480 0.511561

495 0.566115

510 0.609594

525 0.643841

540 0.603648

555 0.642272

570 0.654792

585 0.701644

600 0.687284

615 0.72434

630 0.717342

645 0.757968

660 0.741438

675 0.781113

690 0.775525

705 0.797777

720 0.763684

735 0.799423

750 0.835751

765 0.857211

780 0.830771

795 0.865105

810 0.901918

825 0.906587

840 0.875525

855 0.885894

870 0.933278

885 0.928878

900 0.91649

915 0.931539

930 0.968269

945 0.978404

960 0.97124

975 0.985896

990 1.035

1005 1.01349

1020 1.01274

1035 0.996504

1050 1.07407

1065 1.07833

1080 1.04895

1095 1.05031

1110 1.07853

1125 1.07463

1140 1.06778

1155 1.06506

1170 1.14111

1185 1.12116

1200 1.12392

1215 1.13202

1230 1.24039

1245 1.20093

1260 1.17908

1275 1.19688

1290 1.26105

1305 1.25361

1320 1.23729

1335 1.26239

1350 1.32765

1365 1.28972

1380 1.29537

1395 1.32336

1410 1.41584

1425 1.39145

1440 1.37134

1455 1.42854

1470 1.51323

1485 1.47365

1500 1.46507

1515 1.54754

1530 1.60096

1545 1.55594

1560 1.55877

1575 1.65394

1590 1.73752

1605 1.68334

1620 1.66882

1635 1.76635

1650 1.85833

1665 1.80184

1680 1.78311

1695 1.89976

1710 1.97983

1725 1.92104

1740 1.92269

1755 2.0184

1770 2.10286

1785 2.06626

1800 2.06471

1815 2.18099

1830 2.26553

1845 2.22756

1860 2.22

1875 2.36497

1890 2.46046

1905 2.407

1920 2.3987

1935 2.55776

1950 2.63315

1965 2.59315

1980 2.55419

1995 2.71286

2010 2.78878

2025 2.75738

2040 2.72414

2055 2.88403

2070 2.96616

2085 2.93792

2100 2.90923

2115 3.06273

2130 3.1626

2145 3.11158

2160 3.05308

2175 3.19835

2190 3.29915

2205 3.26802

2220 3.20648

2235 3.38951

2250 3.49579

2265 3.45894

2280 3.40457

2295 3.58563

2310 3.72171

2325 3.70945

2340 3.61252

2355 3.7693

2370 3.86969

2385 3.83928

2400 3.73346

2415 3.89696

2430 3.96833

2445 3.97257

2460 3.86604

2475 4.06129

2490 4.14356

2505 4.11995

2520 4.02763

2535 4.18876

2550 4.29249

2565 4.3155

2580 4.1691

2595 4.34645

2610 4.43975

2625 4.45221

2640 4.3164

2655 4.49446

2670 4.60037

2685 4.60273

2700 4.46259

2715 4.6178

2730 4.76284

2745 4.78594

2760 4.65806

2775 4.7506

2790 4.88175

2805 4.8968

2820 4.68872

2835 4.839

2850 4.92712

2865 4.90952

2880 4.71909

2895 4.92349

2910 5.04185

2925 4.99416

2940 4.7642

2955 5.00985

2970 5.10626

2985 5.09642

3000 4.83395

3015 5.06813

3030 5.16863

3045 5.19252

3060 4.9304

3075 5.01623

3090 5.11426

3105 5.09151

3120 4.82639

3135 4.91651

3150 4.89307

3165 4.89014

3180 4.72012

3195 4.86547

3210 4.92437

3225 4.98333

3240 4.66099

3255 4.82243

3270 4.8064

3285 4.90975

3300 4.6295

3315 4.74915

3330 4.63291

3345 4.69562

3360 4.48

3375 4.70163

3390 4.58711

3405 4.66068

3420 4.56816

3435 4.57367

3450 4.59348

3465 4.78616

3480 4.53201

3495 4.44958

3510 4.24926

3525 4.34915

3540 4.15849

3555 4.26216

3570 3.99578

3585 4.05629

3600 3.82902

3615 4.41253

3630 4.25673

3645 4.35746

3660 4.11095

3675 4.55716

3690 4.31626

3705 4.47436

3720 4.15674

3735 4.63168

3750 4.5088

3765 4.55065

3780 4.29651

3795 4.60189

3810 4.26302

3825 4.37571

3840 4.03421

3855 4.41262

3870 4.07705

3885 4.24498

3900 3.77333

3915 4.82857

3930 4.15663

3945 4.31206

3960 3.84398

3975 4.29754

3990 3.57826

4005 3.52507

4020 3.06567

4035 3.9053

4050 2.97522

4065 2.83015

4080 2.48538

4095 3.2757

4110 3.22741

4125 3.30468

4140 3.22231

4155 3.16853

4170 3.18627

4185 3.3301

4200 3.45643

@ title "Mean Square Displacement"

@ xaxis label "Time (ps)"

@ yaxis label "MSD (nm\S2\N)"

@TYPE xy

# MSD gathered over 3990 ps with 67 restarts

# Diffusion constants fitted from time 405 to 3585 ps

# D[ POT] = 0.4167 (+/- 0.1340) (1e-5 cm^2/s)

0 0

15 0.0356611

30 0.0591923

45 0.0964603

60 0.110835

75 0.143459

90 0.172616

105 0.177618

120 0.171192

135 0.18408

150 0.21624

165 0.231699

180 0.218091

195 0.228254

210 0.241944

225 0.274522

240 0.263715

255 0.293472

270 0.298753

285 0.326638

300 0.311814

315 0.362289

330 0.384934

345 0.396555

360 0.375822

375 0.429809

390 0.441981

405 0.46107

420 0.431101

435 0.465719

450 0.508298

465 0.549376

480 0.533954

495 0.572089

510 0.622898

525 0.666885

540 0.621243

555 0.665948

570 0.677201

585 0.734039

600 0.710507

615 0.758635

630 0.749777

645 0.791305

660 0.769321

675 0.804865

690 0.811013

705 0.848187

720 0.811006

735 0.83444

750 0.874608

765 0.910957

780 0.877183

795 0.90873

810 0.945784

825 0.964806

840 0.920214

855 0.929406

870 0.969865

885 0.978309

900 0.950926

915 0.97033

930 1.0033

945 1.02958

960 1.00876

975 1.02334

990 1.07042

1005 1.06524

1020 1.05096

1035 1.04011

1050 1.11276

1065 1.13503

1080 1.08877

1095 1.09498

1110 1.12156

1125 1.13733

1140 1.11782

1155 1.11322

1170 1.19009

1185 1.18744

1200 1.18059

1215 1.18941

1230 1.30665

1245 1.28423

1260 1.25428

1275 1.25898

1290 1.33427

1305 1.34601

1320 1.32612

1335 1.33282

1350 1.41252

1365 1.37894

1380 1.38751

1395 1.38742

1410 1.50746

1425 1.49033

1440 1.47736

1455 1.49345

1470 1.60846

1485 1.55667

1500 1.56165

1515 1.59777

1530 1.69005

1545 1.62198

1560 1.64643

1575 1.68618

1590 1.81828

1605 1.73822

1620 1.74901

1635 1.78305

1650 1.92749

1665 1.87649

1680 1.88111

1695 1.94466

1710 2.06468

1725 1.99531

1740 2.0187

1755 2.07393

1770 2.20602

1785 2.14451

1800 2.16926

1815 2.23655

1830 2.37946

1845 2.32482

1860 2.3491

1875 2.4147

1890 2.57093

1905 2.49886

1920 2.52444

1935 2.6079

1950 2.7457

1965 2.68247

1980 2.67603

1995 2.76582

2010 2.90942

2025 2.88022

2040 2.8815

2055 2.96291

2070 3.10859

2085 3.09064

2100 3.09477

2115 3.17102

2130 3.33604

2145 3.28692

2160 3.25812

2175 3.3259

2190 3.4988

2205 3.48507

2220 3.45567

2235 3.53878

2250 3.71855

2265 3.72811

2280 3.70513

2295 3.77726

2310 3.98387

2325 4.01228

2340 3.93884

2355 3.98805

2370 4.16453

2385 4.16924

2400 4.09017

2415 4.12652

2430 4.2792

2445 4.35842

2460 4.28071

2475 4.32414

2490 4.48198

2505 4.5266

2520 4.46231

2535 4.47673

2550 4.66474

2565 4.7459

2580 4.62305

2595 4.64707

2610 4.83864

2625 4.94419

2640 4.83922

2655 4.82321

2670 5.02561

2685 5.14654

2700 5.03284

2715 4.99271

2730 5.23622

2745 5.34479

2760 5.24692

2775 5.14045

2790 5.391

2805 5.51972

2820 5.335

2835 5.24838

2850 5.45252

2865 5.37037

2880 5.22292

2895 5.16378

2910 5.45961

2925 5.31444

2940 5.1486

2955 5.1078

2970 5.40022

2985 5.40772

3000 5.20961

3015 5.12442

3030 5.40275

3045 5.40984

3060 5.20172

3075 5.09824

3090 5.39101

3105 5.18921

3120 4.99088

3135 4.90242

3150 5.10036

3165 5.00834

3180 4.95211

3195 4.79163

3210 5.0824

3225 4.85749

3240 4.61472

3255 4.4975

3270 4.75534

3285 4.41422

3300 4.25899

3315 4.16528

3330 4.3519

3345 3.94443

3360 3.90927

3375 3.80861

3390 4.01383

3405 3.99612

3420 4.11558

3435 3.93692

3450 4.23975

3465 4.17121

3480 4.03382

3495 3.96219

3510 4.05034

3525 3.71016

3540 3.70129

3555 3.81214

3570 3.87524

3585 3.36898

3600 3.36135

3615 3.65609

3630 3.92765

3645 3.31243

3660 3.31488

3675 3.53681

3690 3.76727

3705 3.96301

3720 3.83867

3735 4.20002

3750 4.50135

3765 3.44175

3780 3.4387

3795 3.66575

3810 3.96003

3825 3.89561

3840 3.82662

3855 4.12862

3870 4.3712

3885 4.78732

3900 4.31167

3915 5.1373

3930 4.69811

3945 4.77202

3960 4.12435

3975 5.87905

3990 5.72411

@ title "Mean Square Displacement"

@ xaxis label "Time (ps)"

@ yaxis label "MSD (nm\S2\N)"

@TYPE xy

# MSD gathered over 4785 ps with 80 restarts

# Diffusion constants fitted from time 480 to 4305 ps

# D[ POT] = 0.3445 (+/- 0.1801) (1e-5 cm^2/s)

0 0

15 0.0415501

30 0.0660334

45 0.0852122

60 0.109943

75 0.12738

90 0.137818

105 0.139164

120 0.175686

135 0.175008

150 0.164652

165 0.169199

180 0.188818

195 0.218784

210 0.21112

225 0.227671

240 0.237882

255 0.259593

270 0.253272

285 0.296601

300 0.317638

315 0.338414

330 0.33071

345 0.374565

360 0.379698

375 0.407703

390 0.384853

405 0.428245

420 0.421647

435 0.455608

450 0.419085

465 0.46361

480 0.49292

495 0.529693

510 0.484862

525 0.530319

540 0.558677

555 0.593028

570 0.57043

585 0.609671

600 0.629446

615 0.671504

630 0.649175

645 0.66204

660 0.66974

675 0.709069

690 0.671797

705 0.694451

720 0.720004

735 0.753654

750 0.737429

765 0.751416

780 0.792245

795 0.803705

810 0.780378

825 0.772861

840 0.819314

855 0.823751

870 0.798313

885 0.797829

900 0.83755

915 0.847814

930 0.855866

945 0.854117

960 0.917427

975 0.902226

990 0.915725

1005 0.903297

1020 0.983893

1035 0.968693

1050 0.937181

1065 0.926994

1080 0.949897

1095 0.956041

1110 0.95587

1125 0.953287

1140 0.988397

1155 0.978909

1170 0.997584

1185 0.994595

1200 1.09605

1215 1.06999

1230 1.05972

1245 1.05244

1260 1.14304

1275 1.12938

1290 1.11621

1305 1.11678

1320 1.19019

1335 1.15371

1350 1.1548

1365 1.15897

1380 1.24029

1395 1.21918

1410 1.20905

1425 1.22959

1440 1.3035

1455 1.27338

1470 1.26469

1485 1.30375

1500 1.37126

1515 1.32735

1530 1.32118

1545 1.37864

1560 1.45644

1575 1.41762

1590 1.40952

1605 1.45555

1620 1.55387

1635 1.51762

1650 1.50537

1665 1.56471

1680 1.67647

1695 1.63682

1710 1.62159

1725 1.68038

1740 1.77637

1755 1.74282

1770 1.73381

1785 1.80769

1800 1.89982

1815 1.86053

1830 1.85891

1845 1.94181

1860 2.05022

1875 2.01249

1890 1.99954

1905 2.07526

1920 2.20859

1935 2.17522

1950 2.12989

1965 2.2233

1980 2.33457

1995 2.30252

2010 2.27602

2025 2.38122

2040 2.4912

2055 2.46857

2070 2.46269

2085 2.5527

2100 2.6927

2115 2.67179

2130 2.63656

2145 2.71195

2160 2.85169

2175 2.82616

2190 2.75904

2205 2.84881

2220 2.96564

2235 2.94527

2250 2.89576

2265 2.99835

2280 3.12488

2295 3.11941

2310 3.06096

2325 3.14436

2340 3.32454

2355 3.32648

2370 3.26051

2385 3.32373

2400 3.47222

2415 3.46455

2430 3.38581

2445 3.45405

2460 3.56857

2475 3.54276

2490 3.45656

2505 3.51512

2520 3.63238

2535 3.65901

2550 3.55673

2565 3.62372

2580 3.75363

2595 3.79233

2610 3.68064

2625 3.74588

2640 3.84384

2655 3.85782

2670 3.76889

2685 3.82178

2700 3.96516

2715 3.97244

2730 3.87754

2745 3.90281

2760 4.10401

2775 4.14842

2790 4.00105

2805 4.01054

2820 4.19171

2835 4.23772

2850 4.08203

2865 4.10172

2880 4.23944

2895 4.26726

2910 4.1152

2925 4.1652

2940 4.29784

2955 4.29136

2970 4.14464

2985 4.17632

3000 4.37579

3015 4.39111

3030 4.1958

3045 4.22754

3060 4.41082

3075 4.42877

3090 4.19361

3105 4.24942

3120 4.33708

3135 4.35598

3150 4.15614

3165 4.23939

3180 4.35533

3195 4.40541

3210 4.23352

3225 4.2999

3240 4.45657

3255 4.50346

3270 4.30573

3285 4.33425

3300 4.4213

3315 4.49359

3330 4.26429

3345 4.32384

3360 4.38699

3375 4.45663

3390 4.24923

3405 4.36197

3420 4.42166

3435 4.53306

3450 4.37046

3465 4.48832

3480 4.61358

3495 4.76813

3510 4.64963

3525 4.72926

3540 4.76553

3555 4.88506

3570 4.68906

3585 4.80698

3600 4.61617

3615 4.73554

3630 4.58919

3645 4.77792

3660 4.69998

3675 4.81141

3690 4.62935

3705 4.91013

3720 4.83252

3735 4.98109

3750 4.81414

3765 5.07883

3780 4.93746

3795 5.08014

3810 4.87023

3825 5.1347

3840 5.04322

3855 5.11669

3870 4.92237

3885 5.17145

3900 5.0742

3915 5.22842

3930 5.0553

3945 5.27166

3960 5.05639

3975 5.22022

3990 4.99623

4005 5.26584

4020 4.82975

4035 4.98228

4050 4.74252

4065 5.05334

4080 4.73091

4095 4.81381

4110 4.61609

4125 4.81278

4140 4.50846

4155 4.55986

4170 4.42774

4185 4.5751

4200 4.3936

4215 4.51279

4230 4.4567

4245 4.43023

4260 4.11005

4275 4.24712

4290 4.21636

4305 4.19359

4320 4.00093

4335 4.11524

4350 4.05603

4365 4.01135

4380 4.0958

4395 4.20566

4410 4.1871

4425 4.1649

4440 3.80958

4455 3.88454

4470 3.90513

4485 3.87713

4500 3.24454

4515 3.30497

4530 3.28962

4545 3.21342

4560 3.24959

4575 3.28935

4590 3.33756

4605 3.28509

4620 3.3696

4635 3.4528

4650 3.59741

4665 3.49459

4680 3.39207

4695 3.43387

4710 3.71908

4725 3.64833

4740 3.26479

4755 3.65459

4770 4.18391

4785 4.12722

@ title "Mean Square Displacement"

@ xaxis label "Time (ps)"

@ yaxis label "MSD (nm\S2\N)"

@TYPE xy

# MSD gathered over 4695 ps with 79 restarts

# Diffusion constants fitted from time 465 to 4230 ps

# D[ POT] = 1.9169 (+/- 0.7259) (1e-5 cm^2/s)

0 0

15 0.177235

30 0.401828

45 0.602265

60 0.731753

75 0.766645

90 1.0517

105 1.21036

120 1.33435

135 1.32792

150 1.60571

165 1.82012

180 1.97499

195 1.92293

210 2.21461

225 2.4486

240 2.59887

255 2.5476

270 2.75303

285 2.89792

300 3.09553

315 2.92229

330 3.21639

345 3.34726

360 3.40794

375 3.22986

390 3.33733

405 3.41012

420 3.42064

435 3.2115

450 3.38693

465 3.35598

480 3.3926

495 3.2658

510 3.39543

525 3.45211

540 3.51622

555 3.51725

570 3.56375

585 3.57729

600 3.68863

615 3.71353

630 3.96226

645 3.95469

660 4.1636

675 4.24915

690 4.47141

705 4.48274

720 4.75104

735 4.8066

750 5.16965

765 5.16076

780 5.38489

795 5.34723

810 5.76204

825 5.75405

840 5.86801

855 5.83736

870 6.29418

885 6.26505

900 6.47051

915 6.29035

930 6.65807

945 6.62114

960 6.71382

975 6.40593

990 6.74075

1005 6.79176

1020 6.93787

1035 6.64049

1050 7.15869

1065 7.09111

1080 7.20146

1095 6.89011

1110 7.47529

1125 7.50813

1140 7.80908

1155 7.65149

1170 8.17576

1185 8.25935

1200 8.56975

1215 8.37438

1230 8.85733

1245 8.98903

1260 9.26102

1275 9.0928

1290 9.5311

1305 9.68661

1320 10.0909

1335 9.8837

1350 10.3563

1365 10.6125

1380 11.0518

1395 10.7076

1410 11.1093

1425 11.2163

1440 11.8001

1455 11.3775

1470 11.8333

1485 11.9338

1500 12.4675

1515 12.0667

1530 12.4681

1545 12.5535

1560 13.1709

1575 12.6579

1590 13.1569

1605 13.315

1620 13.9116

1635 13.4011

1650 13.8157

1665 13.8874

1680 14.5225

1695 14.1505

1710 14.5754

1725 14.7362

1740 15.3763

1755 15.1704

1770 15.6032

1785 15.6887

1800 16.3255

1815 16.0291

1830 16.4835

1845 16.5157

1860 17.1493

1875 16.8058

1890 17.4168

1905 17.1905

1920 17.8059

1935 17.3719

1950 18.0312

1965 17.743

1980 18.4497

1995 17.9434

2010 18.519

2025 18.0999

2040 18.8163

2055 18.0583

2070 18.5854

2085 18.1461

2100 18.8148

2115 18.1156

2130 18.6712

2145 18.2255

2160 19.0475

2175 18.3853

2190 19.0549

2205 18.6789

2220 19.6232

2235 19.0513

2250 19.5419

2265 19.05

2280 20.0346

2295 19.4132

2310 19.9729

2325 19.542

2340 20.4929

2355 19.9004

2370 20.5938

2385 20.1107

2400 21.1416

2415 20.5234

2430 21.2311

2445 20.8816

2460 21.8923

2475 21.1602

2490 21.5233

2505 21.0595

2520 22.0096

2535 21.6302

2550 21.918

2565 21.6347

2580 22.5446

2595 22.1851

2610 22.4959

2625 22.1692

2640 23.2629

2655 22.8601

2670 23.2315

2685 22.9151

2700 24.1599

2715 23.6838

2730 24.0559

2745 23.4903

2760 24.8116

2775 24.303

2790 24.5586

2805 23.9355

2820 25.3347

2835 24.9359

2850 24.932

2865 24.1785

2880 25.5651

2895 25.1054

2910 24.8101

2925 24.0537

2940 25.2066

2955 25.0973

2970 24.853

2985 23.9459

3000 24.9365

3015 25.3042

3030 25.0797

3045 24.3043

3060 25.2276

3075 25.7239

3090 25.1961

3105 24.2271

3120 25.2071

3135 25.7547

3150 24.7557

3165 23.8413

3180 24.6352

3195 25.3684

3210 24.4832

3225 23.4127

3240 24.321

3255 25.041

3270 24.4162

3285 23.4139

3300 24.3224

3315 24.8374

3330 24.693

3345 23.8037

3360 24.6114

3375 24.86

3390 24.6468

3405 23.8086

3420 24.4227

3435 25.0494

3450 25.5827

3465 24.9105

3480 25.4312

3495 26.0817

3510 26.1729

3525 25.4129

3540 25.7883

3555 27.0145

3570 27.0033

3585 26.0348

3600 26.3558

3615 27.8235

3630 26.8325

3645 25.9027

3660 26.0749

3675 27.6478

3690 26.2614

3705 25.4822

3720 25.982

3735 27.3666

3750 26.2522

3765 25.8088

3780 26.3695

3795 27.704

3810 25.6064

3825 25.5324

3840 26.2186

3855 27.5751

3870 26.1231

3885 26.6323

3900 27.2713

3915 28.8892

3930 27.687

3945 28.3773

3960 29.4362

3975 30.965

3990 29.4857

4005 30.1434

4020 31.1238

4035 33.1893

4050 32.0832

4065 32.4452

4080 33.4318

4095 35.7353

4110 33.0843

4125 33.4562

4140 34.3296

4155 36.6757

4170 32.9728

4185 32.7379

4200 33.7371

4215 35.6268

4230 28.6863

4245 28.5814

4260 28.1982

4275 28.3972

4290 23.7343

4305 23.5083

4320 24.4563

4335 26.4916

4350 20.8058

4365 20.7131

4380 21.1931

4395 23.148

4410 19.5316

4425 18.5087

4440 19.1712

4455 21.2045

4470 17.4425

4485 16.531

4500 16.5267

4515 19.4228

4530 19.789

4545 17.9687

4560 18.515

4575 21.1741

4590 20.1825

4605 17.0656

4620 16.37

4635 17.4855

4650 19.1485

4665 14.3382

4680 14.1803

4695 14.5595

@ title "Mean Square Displacement"

@ xaxis label "Time (ps)"

@ yaxis label "MSD (nm\S2\N)"

@TYPE xy

# MSD gathered over 3810 ps with 67 restarts

# Diffusion constants fitted from time 405 to 3585 ps

# D[ POT] = 2.0589 (+/- 1.0191) (1e-5 cm^2/s)

0 0

15 0.195356

30 0.398165

45 0.57228

60 0.765789

75 0.938621

90 1.08547

105 1.1797

120 1.31198

135 1.51164

150 1.69882

165 1.76416

180 1.87081

195 2.11776

210 2.29801

225 2.25122

240 2.27751

255 2.53778

270 2.56343

285 2.60982

300 2.74429

315 3.11552

330 3.21884

345 3.28249

360 3.37723

375 3.61603

390 3.68123

405 3.80011

420 3.78289

435 4.05032

450 4.11857

465 4.26521

480 4.14477

495 4.26815

510 4.38966

525 4.74767

540 4.59483

555 4.82879

570 4.94069

585 5.39136

600 5.32423

615 5.51737

630 5.62183

645 6.06879

660 6.08965

675 6.20335

690 6.24617

705 6.60369

720 6.56937

735 6.71516

750 6.81495

765 7.28459

780 7.34523

795 7.44181

810 7.50723

825 8.07369

840 7.83643

855 7.92055

870 8.11463

885 8.69381

900 8.52891

915 8.67925

930 8.77249

945 9.42023

960 9.33226

975 9.36147

990 9.55

1005 10.1988

1020 10.1405

1035 10.1983

1050 10.4154

1065 11.1144

1080 11.0875

1095 11.0487

1110 11.203

1125 12.0341

1140 12.0616

1155 12.2104

1170 12.4628

1185 13.1082

1200 13.1418

1215 13.3564

1230 13.5715

1245 14.3236

1260 14.2506

1275 14.5378

1290 14.8027

1305 15.6018

1320 15.3542

1335 15.6762

1350 15.7623

1365 16.6946

1380 16.3867

1395 17.0283

1410 17.0141

1425 17.9201

1440 17.4118

1455 17.8793

1470 17.9675

1485 18.8708

1500 18.4854

1515 18.741

1530 18.7159

1545 19.799

1560 19.4864

1575 19.4193

1590 19.3889

1605 20.5915

1620 20.4674

1635 20.7959

1650 20.4374

1665 22.0773

1680 22.1187

1695 22.4955

1710 22.1075

1725 23.4775

1740 23.4618

1755 23.912

1770 23.5097

1785 25.0298

1800 24.766

1815 24.8449

1830 24.458

1845 26.0781

1860 25.8634

1875 26.1516

1890 25.3927

1905 26.9111

1920 26.8837

1935 27.2233

1950 26.1209

1965 27.9721

1980 27.5763

1995 27.6458

2010 26.4154

2025 28.0939

2040 27.5491

2055 27.0998

2070 25.7893

2085 27.5567

2100 26.9295

2115 26.6919

2130 25.354

2145 27.1544

2160 26.8411

2175 27.1237

2190 25.4126

2205 27.0141

2220 26.9046

2235 27.2752

2250 26.0826

2265 27.888

2280 27.7954

2295 27.737

2310 26.5702

2325 27.8127

2340 27.2092

2355 27.6154

2370 26.6088

2385 28.3002

2400 27.6637

2415 28.3018

2430 27.057

2445 28.6137

2460 28.1045

2475 29.1454

2490 27.7252

2505 29.5233

2520 29.1851

2535 30.0114

2550 28.8119

2565 30.5088

2580 30.2239

2595 30.7001

2610 29.3918

2625 30.6866

2640 30.707

2655 31.2167

2670 29.6987

2685 31.1607

2700 31.2108

2715 31.7915

2730 29.2527

2745 30.7281

2760 31.2438

2775 32.4696

2790 29.9377

2805 31.3212

2820 31.5863

2835 32.6134

2850 29.9974

2865 31.073

2880 31.2328

2895 32.1908

2910 29.4118

2925 30.5029

2940 30.4806

2955 31.2538

2970 28.7591

2985 30.2267

3000 30.5054

3015 30.223

3030 27.4492

3045 28.6889

3060 28.762

3075 28.4727

3090 26.2781

3105 28.5857

3120 29.2347

3135 28.5294

3150 25.3299

3165 26.5757

3180 26.5125

3195 25.4991

3210 23.0989

3225 24.8006

3240 25.046

3255 25.2089

3270 23.0925

3285 24.1251

3300 24.6193

3315 24.5701

3330 22.7642

3345 24.9411

3360 25.7964

3375 25.7886

3390 24.5433

3405 25.0547

3420 25.5891

3435 25.0893

3450 23.2614

3465 25.3624

3480 25.5047

3495 25.7135

3510 23.7849

3525 26.6821

3540 27.3781

3555 26.9671

3570 25.06

3585 26.7013

3600 27.8748

3615 27.7982

3630 26.1628

3645 26.4908

3660 28.5668

3675 27.2509

3690 27.0742

3705 26.7805

3720 27.9309

3735 27.7647

3750 27.3136

3765 28.3718

3780 29.8636

3795 30.8007

3810 32.3191

@ title "Mean Square Displacement"

@ xaxis label "Time (ps)"

@ yaxis label "MSD (nm\S2\N)"

@TYPE xy

# MSD gathered over 4395 ps with 74 restarts

# Diffusion constants fitted from time 435 to 3960 ps

# D[ POT] = 0.3346 (+/- 0.2735) (1e-5 cm^2/s)

0 0

15 0.0400313

30 0.0666191

45 0.100898

60 0.130118

75 0.150015

90 0.167724

105 0.20253

120 0.200541

135 0.190324

150 0.201446

165 0.22196

180 0.23739

195 0.229287

210 0.24827

225 0.256353

240 0.285959

255 0.282446

270 0.32529

285 0.322915

300 0.359142

315 0.330312

330 0.399635

345 0.410834

360 0.442938

375 0.420183

390 0.482409

405 0.481399

420 0.520759

435 0.47326

450 0.532596

465 0.553845

480 0.584029

495 0.541135

510 0.607253

525 0.632213

540 0.673776

555 0.65164

570 0.697201

585 0.718071

600 0.755225

615 0.744527

630 0.7708

645 0.777395

660 0.802521

675 0.761003

690 0.802087

705 0.823182

720 0.852825

735 0.805278

750 0.841904

765 0.869801

780 0.902819

795 0.883592

810 0.917373

825 0.941878

840 0.961496

855 0.933392

870 0.950638

885 0.963666

900 0.968752

915 0.965036

930 0.970616

945 0.993702

960 1.0124

975 1.01199

990 1.01569

1005 1.06284

1020 1.03823

1035 1.01745

1050 1.01169

1065 1.06136

1080 1.06755

1095 1.04544

1110 1.05483

1125 1.07547

1140 1.06312

1155 1.0735

1170 1.06654

1185 1.14885

1200 1.12025

1215 1.11544

1230 1.121

1245 1.22141

1260 1.19095

1275 1.16616

1290 1.18687

1305 1.25385

1320 1.23248

1335 1.21326

1350 1.24045

1365 1.32291

1380 1.28584

1395 1.27437

1410 1.31337

1425 1.37702

1440 1.35481

1455 1.33433

1470 1.40375

1485 1.44657

1500 1.40844

1515 1.42283

1530 1.50211

1545 1.57114

1560 1.52779

1575 1.52199

1590 1.58909

1605 1.67401

1620 1.64732

1635 1.60857

1650 1.69203

1665 1.77129

1680 1.73846

1695 1.71581

1710 1.81551

1725 1.89533

1740 1.85295

1755 1.85656

1770 1.93872

1785 2.00909

1800 1.95986

1815 1.96165

1830 2.08538

1845 2.15627

1860 2.10718

1875 2.10586

1890 2.23389

1905 2.32927

1920 2.27013

1935 2.24832

1950 2.37239

1965 2.44704

1980 2.40966

1995 2.36587

2010 2.53454

2025 2.59739

2040 2.55669

2055 2.5545

2070 2.69849

2085 2.79934

2100 2.76635

2115 2.74476

2130 2.86236

2145 2.96374

2160 2.91751

2175 2.85237

2190 2.99138

2205 3.06839

2220 3.03439

2235 2.97737

2250 3.16086

2265 3.23738

2280 3.22136

2295 3.18663

2310 3.33746

2325 3.45335

2340 3.4473

2355 3.37215

2370 3.51854

2385 3.60172

2400 3.56509

2415 3.51052

2430 3.64612

2445 3.75397

2460 3.70691

2475 3.6207

2490 3.75974

2505 3.89082

2520 3.8468

2535 3.73412

2550 3.89307

2565 4.01029

2580 4.0038

2595 3.87088

2610 4.05746

2625 4.16003

2640 4.15063

2655 4.04474

2670 4.21552

2685 4.32113

2700 4.30223

2715 4.19835

2730 4.36358

2745 4.50637

2760 4.52564

2775 4.40031

2790 4.53653

2805 4.6186

2820 4.63896

2835 4.48106

2850 4.62303

2865 4.69393

2880 4.73567

2895 4.54926

2910 4.75888

2925 4.80391

2940 4.80816

2955 4.63667

2970 4.85153

2985 4.91659

3000 4.92042

3015 4.74767

3030 4.89951

3045 5.04511

3060 5.04327

3075 4.87672

3090 4.85364

3105 5.0198

3120 5.00453

3135 4.75883

3150 4.90548

3165 4.96674

3180 4.98877

3195 4.78495

3210 4.99612

3225 5.06691

3240 5.07488

3255 4.77912

3270 5.01954

3285 5.0169

3300 5.02895

3315 4.75641

3330 4.98068

3345 4.94874

3360 4.92451

3375 4.66419

3390 4.80248

3405 4.80408

3420 4.8193

3435 4.53854

3450 4.51848

3465 4.54978

3480 4.68377

3495 4.40931

3510 4.36902

3525 4.29244

3540 4.36453

3555 4.1544

3570 4.19572

3585 4.06463

3600 4.16889

3615 4.04365

3630 4.0461

3645 4.00162

3660 4.11674

3675 3.84832

3690 3.84736

3705 3.56456

3720 3.67387

3735 3.47539

3750 3.66529

3765 3.48028

3780 3.55456

3795 3.35991

3810 3.57876

3825 3.36598

3840 3.43273

3855 3.2151

3870 3.47307

3885 3.34923

3900 3.46957

3915 3.23378

3930 3.74815

3945 3.67981

3960 3.74273

3975 3.54355

3990 3.54138

4005 3.31333

4020 3.46003

4035 3.16576

4050 3.7451

4065 3.38931

4080 3.54634

4095 3.09177

4110 4.14722

4125 3.44641

4140 3.55977

4155 3.08188

4170 4.10935

4185 3.40448

4200 3.35007

4215 2.91227

4230 3.66849

4245 2.83504

4260 2.71923

4275 2.42417

4290 2.80664

4305 2.76452

4320 2.83919

4335 2.77344

4350 2.40868

4365 2.42703

4380 2.55412

4395 2.66445
